# Artificial Piezoelectric Interface Enables Ultrafast Interfacial Ion Kinetic for Highly‐Sensitive Piezoionic Sensors

**DOI:** 10.1002/advs.74757

**Published:** 2026-03-10

**Authors:** Yanyu Chen, Xingyue Ling, Rizhong Gao, Xiaohong Zhang, Xi Chen, Chao Lu

**Affiliations:** ^1^ College of Chemistry Chemical Engineering and Materials Science Soochow University Suzhou Jiangsu China; ^2^ Institute of Functional Nano & Soft Materials Soochow University Suzhou Jiangsu China; ^3^ Department of Earth and Environmental Engineering Columbia University New York New York USA

**Keywords:** built‐in electric field, high sensitivity, interfacial ion kinetic, piezoelectric interface, wearable application

## Abstract

Flexible piezoionic sensors are promising in artificial intelligence and biomedical engineering, while presenting low signal, delayed response, and limited sensitivity. This is mainly because the ion migration kinetics is sluggish at electrode/electrolyte interface inside all‐solid‐state piezoionic sensors during the sensing process. Here, an artificial piezoelectric interface is engineered into flexible sensors to enable systematic regulation of piezoionic response behavior. The built‐in electric field based on the piezoelectric interface layer significantly promotes ion migration and strengthens charge enrichment at the electrode/electrolyte interface. As a result, the piezoionic sensor achieves a 16‐fold enhancement of sensing voltage, and delivers excellent performances over previously‐reported similar flexible sensors, such as rapid response and recovery time, high conductivity, excellent linearity, and good durability. Furthermore, wearable applications of piezoionic sensors are demonstrated in various scenarios, including human motion monitoring and human‐machine interaction. This study presents an insight into interface engineering in advancing flexible electrochemical devices.

## Graphical abstract

This study proposes a piezoelectric interface modification strategy to amplify the piezoionic effect. The piezoelectric interface generates an intrinsic electric field, which not only drives rapid ion migration but also concentrates polarized ions on the interface. The flexible sensor delivers superior performance, such as a quick response rate, strong long‐term stability, and high signal linearity.

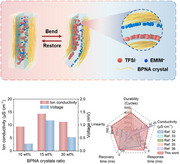

## 1 Introduction

In the era of rapid advancement in artificial intelligence, flexible electronics have emerged as a promising platform for cutting‐edge applications such as robotics [1, 2], healthcare monitoring [3, 4], and human‐machine interfaces [5, 6], owing to their exceptional mechanical adaptability. As essential components for capturing environmental and physiological signals, flexible sensors critically influence the accuracy and reliability of these systems. Particularly, piezoionic sensors have gained significant attention as a promising solution for these applications due to their self‐powered operation, ultra‐thin structure, high detection sensitivity, and stable electrical output [7, 8, 9]. The piezoionic effect utilizes ions as carriers of charge and energy to enable the conversion of mechanical energy into electrical energy. When subjected to external physical stimuli, the internal anions and cations undergo directional migration, resulting in a redistribution of charges and ultimately the generation of voltage signals. In this process, ion transport behavior governs the performance of piezoionic sensors, which is determined by factors including ion size, charge quantity, polarity strength, and electrolyte structure [10, 11]. Therefore, how to optimize the piezoionic effect to enhance voltage output has become a critical scientific challenge and a pivotal step toward the practical implementation of piezoionic sensors.

To enhance the response performance of piezoionic sensors, current research primarily focuses on three key strategies. First, accelerating ion migration rates enables higher transient voltage output. For instance, Deng et al., employed directional freezing technology to construct highly efficient ion transport channels, facilitating rapid ion conduction [12]. Existing studies have also shown that optimizing the polymer matrix structure, such as increasing porosity can accelerate ion migration and significantly increase piezoeionic output [13, 14]. Second, enlarging the mobility difference between cations and anions amplifies the piezoionic effect. This can be achieved through introducing the interaction between specific ions and polymer matrix [15], or by designing channels tailored to the size of specific ions to achieve selective ion transport [16, 17]. Recent study has further demonstrated that the formation of stable coordination bonds within metal ions coordination hydrogel effectively immobilizes metal cations, thus promoting selective and directional diffusion of ligand anions [18]. Third, reducing the initial capacitance of piezoionic sensor without external stimuli helps preserve a wider dynamic range for capacitance variation triggered by external stimuli. Some studies leveraged reversible interactions between fillers and ions, including hydrogen bonds, ionic bonds, and *π*–*π* interaction, to fix some free ions on filler surfaces or within the polymer matrix network, thereby effectively suppressing the initial capacitance [19, 20]. Although these strategies have improved piezoionic performance to some extent, the enhancement remains limited. The primary reason lies in neglecting the regulatory potential inherent in sensor interfaces, such as electrolyte‐electrode interface and interfaces between different electrolyte layers. At the electrode interface of piezoionic sensor, the proportion of polarized ions generated by external stimuli is relatively low. And due to their free mobility, they fail to establish a stable and effective charge accumulation state, resulting in low electrical output that hinders many practical applications. In recent years, increasing attention has been directed toward interfacial regulation: Kim et al., enhanced ion accumulation at the interface by constructing a bilayer piezoionic structure [21], while Yang et al., regulated the formation and self‐assembles of piezoelectric glycine crystals by introducing a PVA‐glycine interface [22]. Therefore, there is an urgent need to establish a general principle and a scalable interfacial structure design to enhance the electrical output, promoting a breakthrough overall piezoionic sensing performance.

Here, we present a piezoionic sensor based on piezoelectric interface engineering, where the piezoelectric interface is composed of BPNA crystals and PEO. The BPNA crystals possess excellent mechanical and piezoelectric properties, capable of withstanding repeated bending deformations without damage. Their non‐polarized piezoelectric coefficient is approximately 7–8 pC/N. The piezoelectric interfaces generate an intrinsic electric field within the piezoionic layer, significantly accelerating ion migration while immobilizing polarized ions at the interfaces between the piezoelectric layer and piezoionic layer, thereby enhancing interfacial ion accumulation and ultimately boosting the output voltage. Therefore, the fabricated sensor achieves an output voltage 16 times higher than that of the unmodified sensor, with an ionic conductivity of up to 14.4 µS/cm. It demonstrates high sensitivity, excellent linearity, and a fast response time of 40 ms. Moreover, the sensor has excellent stability and can endure more than 4000 cycles of significant bending deformation. Owing to such outstanding sensing performance, the sensor enables real‐time monitoring of diverse human physical and physiological signals, ranging from large‐scale joint movements to subtle skin strains. It can accurately distinguish five distinct eye movement positions, showing promising potential for wearable eye‐tracking devices and efficient human‐computer interaction. Additionally, we show its application in monitoring respiration, swallowing, and coughing, where the cross‐validation of these signals facilitates a more accurate assessment of respiratory disorders. The piezoelectric interface engineering strategy proposed in this work offers an effective approach for enhancing piezoionic sensor performance, and provides a solid foundation for future practical applications.

## 2 Results and Discussion

### 2.1 Structural and Morphological Characterization

**FIGURE 1:**
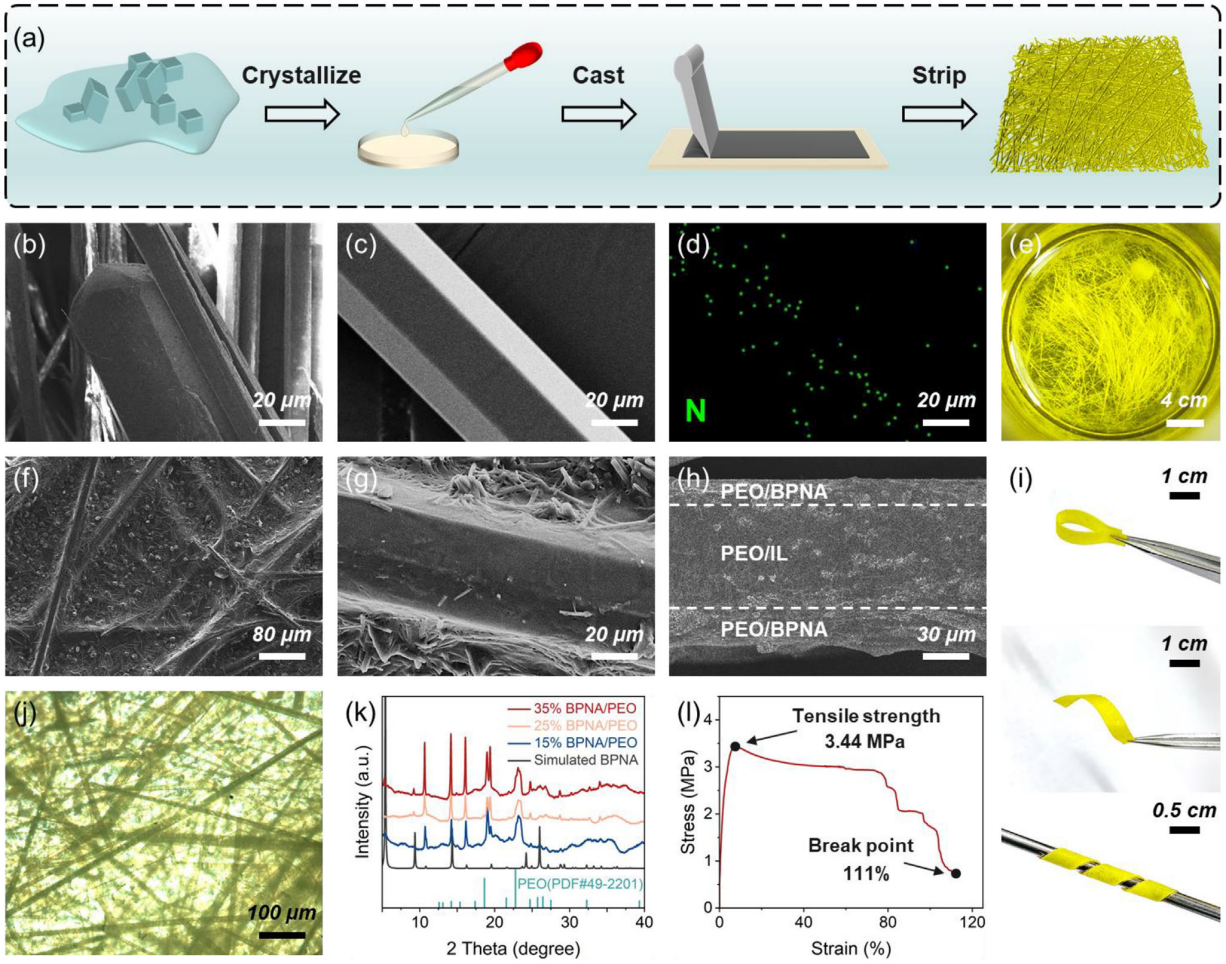
Preparation and characterization of the BPNA/IL/BPNA composite film. (a) Schematic illustration of preparing the BPNA/IL/BPNA composite film. (b,c) SEM images showing hexagonal morphology of the BPNA crystal. (d) EDS image of the element N in the BPNA crystal. (e) Optical image showing yellow needle‐shaped BPNA crystals in a sample bottle. SEM images showing (f) surface morphology, (g) hexagonal morphology of the BPNA crystal, and (h) cross‐section of the BPNA/IL/BPNA composite film. (i) Optical images showing the BPNA/IL/BPNA composite film with excellent flexibility under bending and twisting conditions. (j) HSPM image of the BPNA/IL/BPNA composite film. (k) XRD patterns of the BPNA/IL/BPNA composite films containing different weight contents of BPNA crystals. (l) Strain–stress curve of the BPNA/IL/BPNA composite film with a BPNA crystal mass ratio of 15%.

In order to sense bending deformation effectively, our sensor based on the BPNA/IL/BPNA composite film combines the piezoelectric effect and piezoionic effect, significantly different from conventional piezoionic devices. The BPNA/IL/BPNA composite film can be prepared by a simple layer‐by‐layer solution casting method, and the specific preparation process is illustrated in Figure 1a. Through liquid‐liquid diffusion from a DMF/ethanol solution, yellow needle‐shaped BPNA crystals are successfully obtained (Figure 1e; Figure S1). BPNA crystals exhibit a composite crystal shape consisting of a central hexagonal prism (Figure 1c; Figure S3a–c) and two hexagonal pyramids at both ends sharing their bases with the prism (Figure 1b). The long‐needle‐shaped hexagonal prism morphology is highly consistent with the findings reported earlier [23]. Energy dispersive spectroscopy (EDS) elemental mapping displayed in Figure 1d shows the homogenous distribution of the element N in the needle‐shaped crystal, illustrating the successful cultivation of BPNA crystals. Figures S2 and S3d illustrate the morphology of BPNA crystals under bending states, while Figure 1f shows the bent morphology of crystals within the PEO/BPNA film, demonstrating the excellent elastic bending performance of BPNA crystals. Movie S1 captures the process of applying bending deformation to the BPNA crystal using tweezers. The needle‐shaped crystal can be bent into a closed loop and rapidly return to its original state upon removing external force. Moreover, BPNA crystals are capable of enduring multiple bending‐straightening cycles (Movie S2), effectively preventing piezoelectric performance degradation caused by crystal fracture during practical applications. This behavior originates from the unique spring‐like helical network structure of BPNA crystals, which integrates excellent mechanical flexibility and good resistance to brittle fracture into electronic materials. The presence of numerous well‐defined crystals within the PEO/BPNA films further supports the unique hexagonal prism morphology of BPNA crystals (Figure 1g; Figure S3e,f). The cross‐sectional SEM image of the composite film in Figure 1h confirms the interface modification of PEO/BPNA on the PEO/IL layer, and indicates the film thickness of approximately 120 µm. The image in Figure 1j obtained from the hot‐stage polarizing light microscope (HSPM) indicates that BPNA crystals are uniformly distributed in the composite film. Figures S4 and S5 illustrate the degradation behavior of the composite films in aqueous solution under ambient temperature conditions. The films exhibit excellent solubility and degradability, and the organic crystals can be easily recovered using common, environmentally benign solvents such as acetone. This environmentally friendly strategy prevents the accumulation of non‐degradable residues in electronics, offering a feasible solution to reduce electronic waste.

Upon the analysis and comparison of the XRD patterns (Figure 1k), it can be found that the composite films exhibit distinct characteristic peaks at 10.815°, 14.323°, 16.253°, and 19.563°. These peaks are highly consistent with the simulated XRD pattern of BPNA crystals [23]. And with the increasing content of BPNA crystals, the intensity of the characteristic peaks shows a gradually increasing trend. Figure S6a presents the XRD pattern of BPNA crystal powder, further verifying the phase purity and structural integrity of cultivated BPNA crystals. In addition, compared with the characteristic peaks (18.626° and 22.783°) in the standard XRD pattern of PEO, the characteristic peaks of PEO within the composite films undergo a shift toward higher diffraction angles. This phenomenon can be attributed to the introduction of EMIMTFSI, which modifies the crystalline phase structure of PEO and promotes the transformation toward a more amorphous phase [24]. As the content of ionic liquid increases, the crystallinity of PEO gradually decreases (Figure S6b).

To better apply the composite film, we conduct a detailed analysis of its mechanical properties. As shown in Figure 1i, the BPNA/IL/BPNA composite film exhibits excellent flexibility and toughness, performing well under both bending and twisting conditions. Taking the composite film with a BPNA crystal mass ratio of 15% as an example (Figure 1l), it remains within the elastic deformation range when the strain is less than 8%, fully recovering its original shape upon removal of external forces. An ultimate tensile strength of 3.44 MPa and an elongation at break of 111% can be achieved. Compared with other PEO films doped with organic crystals, the mechanical properties of the BPNA/IL/BPNA composite film have been significantly enhanced [25]. Cracks begin to form and propagate only when the strain reaches 59% (Figure S7), which is attributed to the good viscoelastic properties and the interlayer connection effect of PEO. Additionally, the stress–strain curve shows multiple platforms in the fracture section because of the multilayer structure of the BPNA/IL/BPNA composite film. When the crystal content increases to 25 wt.%, the composite film achieves a tensile strength of 4.52 MPa, but its stretchability and toughness evidently decrease (Figure S8).

### 2.2 Principle and Electrical Sensing Properties

**FIGURE 2:**
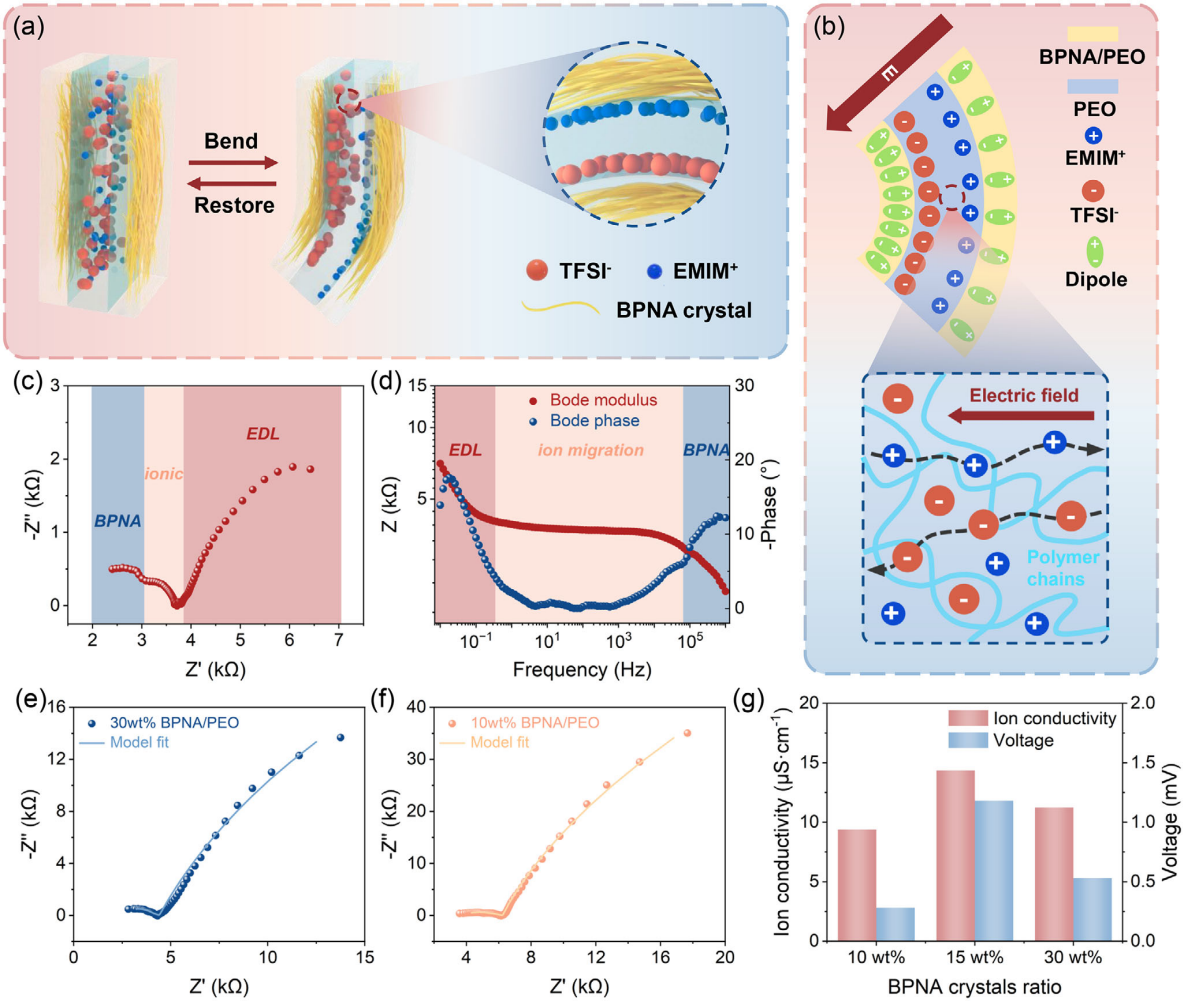
Principle of the piezoionic sensor based on BPNA/IL/BPNA composite film. (a) Schematic illustration of the sensor in bending and restoring states. (b) Schematic illustrating the comprehensive working mechanism of the piezoelectric and piezoionic effect for the sensor. EIS analysis including (c) Nyquist plot and (d) Bode plot for the sensor based on the 15 wt.% BPNA/IL/BPNA composite film. Nyquist plots and fitted curves for the sensor with (e) 30 wt.% and (f) 10 wt.% BPNA crystal content. (g) Ion conductivity and voltage signals of sensors with different weight contents of BPNA crystals.

Figure 2a illustrates the crystal morphology and ion distribution within the sensor based on BPNA/IL/BPNA composite film with and without bending deformation. When subjected to bending stimulation, BPNA crystals undergo deformation, and the cooperative reorientation of molecular dipoles in crystals induces significant net polarization, resulting in piezoelectric voltage. Simultaneously, cations and anions of the ionic liquid migrate in response to the bending stimulus, leading to ion redistribution and consequently generating a voltage. The sensor in our study integrates the piezoionic effect with the piezoelectric effect, aiming to form an internal electric field through charge differences at the two contact interfaces between the PEO/BPNA layer and the PEO/IL layer. The internal electric field is expected to promote ionic migration, amplifies ionic polarization, and ultimately increase the output piezoionic voltage (Figure 2b). Under dynamic bending conditions, the dipole moment of BPNA crystals undergoes reversible reorientation in response to the magnitude and direction of the applied bending strain, thereby sustaining its facilitation of ion migration. In the BPNA crystal, the amino group (donor) and the nitro group (acceptor) are located at the para position of the conjugated benzene ring, contributing to significant polarization (Figure S9). The piezoelectric coefficient of the non‐polar, bulk BPNA crystal is approximately 7–8 pC/N [23]. The voltage output signals of a single BPNA crystal are tested, with each bending deformation displacement controlled at 2 mm, resulting in a generated voltage exceeding 10 mV (Figure S10 and Movie S2).

To investigate the ion dynamics within the interfacial modified PEO/IL layer, we characterize the impedance of sensors under bending stimulation using electrochemical impedance spectroscopy (EIS). Figure 2c,d show the Nyquist plot and Bode plot of the device with a crystal content of 15 wt.%. Based on the varying alternative current frequencies, the Bode modulus curve can be divided into three parts: the diagonal dominated by the electric double layer (EDL) in the low‐frequency region, the flat line dominated by ion migration in the mid‐frequency region, and the diagonal dominated by molecular polarization in the high‐frequency region [26]. This corresponds one‐to‐one to the three semi‐circular regions in the Nyquist plot, respectively representing the PEO/BPNA bulk film, the PEO/IL bulk film, and the accumulated ions at the interfaces (EDL). Therefore, we constructed an equivalent circuit model (Figure S11), where Rs denotes the ohmic resistance of the electrode, R‐BPNA denotes the bulk resistance of the PEO/BPNA layer, R‐ionic denotes the bulk resistance of the PEO/IL layer, which is also be regarded as the ionic resistance [27, 28], and R‐EDL denotes the EDL resistance. CPE‐BPNA and CPE‐ionic are constant phase elements corresponding to their bulk capacitances, while CPE‐EDL is the constant phase element corresponding to the EDL capacitance caused by ionic conduction.

We fit the EIS data with the equivalent circuit model. As shown in Figure 2e,f, and Figure S12, the fitted curves are highly consistent with the original spectrums, verifying the accuracy of the model. Detailed data are provided in Table S1. With the increase in BPNA crystal content, under the same ionic concentration, the ionic resistance is observed to decrease progressively, reaching its minimum at 15 wt.%. Beyond this concentration, the ionic resistance increased from 606 Ω (at 15 wt.%) to 784 Ω (at 30 wt.%). This may be attributed to the agglomeration of polar crystallites at higher concentrations, resulting in the randomization of the dipoles responsible for their piezoelectric response. The internal electric field intensity also weakens, thereby reducing the promoting effect on the ion migration movement. The maximum Q‐EDL value is at 15 wt.%, indicating that the EDL region has high ion conductivity and significant ion accumulation. The ionic conductivities for devices with BPNA crystal contents of 10, 15, and 30 wt.% are 9.39, 14.4, and 11.2 µS/cm, respectively, with corresponding voltage outputs of 0.28, 1.18, and 0.53 mV (Figure 2g). Consequently, when subjected to bending stimulation, the PEO/BPNA layer undergoes piezoelectric polarization, establishing an internal electric field in the PEO/IL layer. Ions respond to the internal electric field, accelerating ion migration and accumulating more ions in EDL, resulting in enhanced output voltage.

**FIGURE 3:**
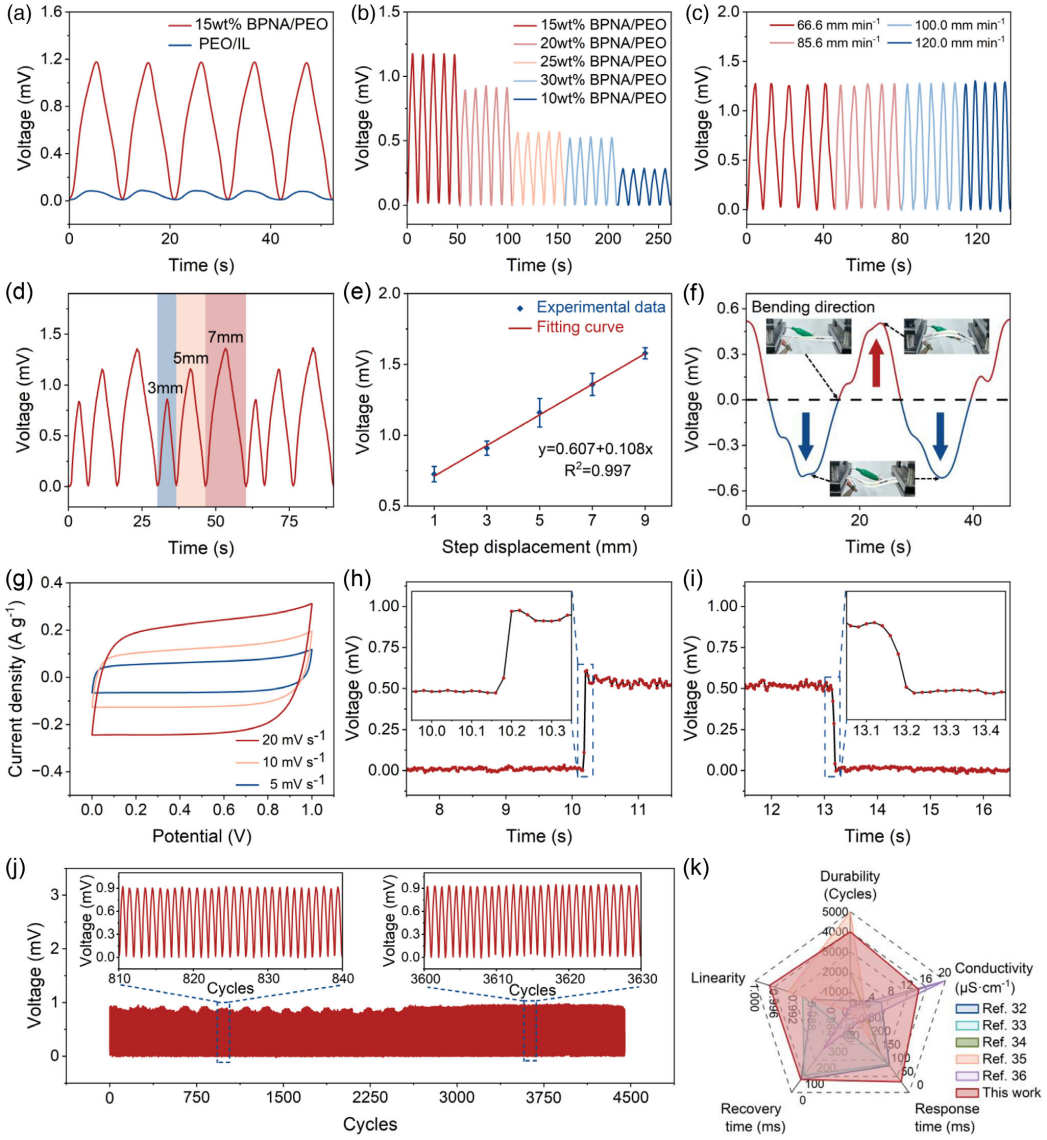
Piezoionic sensing performance of sensors based on BPNA/IL/BPNA composite film. (a) Comparison of voltage signals between PEO/IL piezoionic sensors with 15 wt.% BPNA/PEO interface modification (red line) and without interface modification (blue line). (b) Comparison of voltage signals between sensors with different BPNA crystal contents in interface modification. (c) Sensing response under testing speeds from 33.3 to 60 mm min^−1^. (d) Sensing response to continuous bending deformation of different displacements. (e) Experimental data along with the corresponding fitting curve for repeated signals of the sensor under various bending deformations. Error bars correspond to the standard deviation, where *n* = 5. (f) Sensing response to different bending directions. (g) CV curves of the sensor at different scan rates. (h) Response time and (i) recovery time of the sensor under rapid bending deformation. (j) Durability test results under repetitive deformation. (k) Comprehensive comparison of durability, conductivity, response time, recovery time, and linearity.

Owing to the high piezoelectric coefficient of BPNA crystals, enhanced ionic conductivity, increased capacitance, the optimized device performance shows significant improvement. As shown in Figure 3a, the output voltage of the optimal proportion sensor is 16 times higher than that of the PEO/IL sensor without interface modification (0.073 mV). Figure 3b further presents the voltage response trend of piezoionic sensors with different crystal contents. Compared with the voltage response of piezoelectric PEO/BPNA sensors in Figure S13, it indicates the introduction of a piezoelectric interface enhances the piezoionic effect and consequently increases the output voltage. Under identical bending strain conditions, the sensor exhibits excellent repeatability and stability across a range of testing rates (Figure 3c), verifying its reliable response characteristics in dynamic deformation monitoring. In addition, the optimal sensor enables continuous dynamic recognition of varying displacements and demonstrates an excellent linear relationship between displacement distance and output voltage (Figure 3d,e). As shown in Figure S14, the output voltage also exhibits a strong linear dependence on the applied bending strain within the elastic regime. The bending strain is approximately analyzed based on the Euler–Bernoulli beam theory [29], and the obtained bending curvature is basically consistent with the curvature value extracted from the optical images in the experiment (Figure S15). Beyond this elastic threshold, the voltage signal under plastic deformation exhibits marked degradation (Figure S16), showing a decline in voltage with increasing loading duration. When subjected to identical bending stimuli from both the top and bottom directions, the sensor can output voltage signals with equal magnitude but opposite polarity (Figure 3f). This indicates that the sensor can accurately identify the direction of motion and precisely measure the amplitude of motion without additional signal calibration, thereby offering a substantial advantage for practical applications.

The sensor also displays the typical rectangular cyclic voltammetry curves (Figure 3g) and symmetrical triangular charge–discharge curve (Figure S17), confirming the capacitive nature of the ion migration process. As shown in Figure 3h,i, the sensor exhibits superior sensing performance in terms of response time, with a response time of 40 ms and a recovery time of 80 ms. To further evaluate the rapid response characteristic, balls of 10 and 40 g mass are separately dropped from a height of 10 mm onto the sensor surface and then rebound. As shown in Figure S18, the response time is consistently 10 ms for both impact conditions, the recovery time is 10 ms for the 10 g ball and 20 ms for the 40 g ball. Across a range of external mechanical stimuli, the sensor consistently exhibits outstanding rapid response characteristics. Furthermore, the sensor shows high stability during repeated use. As shown in Figure 3j, the output voltage attenuation remains below 7% after 4000 cycles of testing under a 5 mm displacement bending deformation condition. A comparative stability test on the same sensor under ambient air conditions and controlled humidity conditions is also conducted (Figure S19). At the relative humidity (RH) of 57%, the voltage output increases by 3.4% relative to the ambient air condition (33% RH), and remains stable over 24 h of continuous exposure. This enhancement arises from an appropriate amount of water adsorption, which increases electrolyte conductivity and facilitates ion migration [30, 31]. In a high humidity environment with 85% RH, the sensor shows rapid performance degradation, output voltage dropping by 5.2% after 1 h and by 39.6% after 8 h. Such deterioration arises from excessive water absorption, leading to electrolyte swelling and even decomposition. Therefore, this sensor exhibits excellent stability in human‐comfortable humidity environments, but it should not be used in continuous exposure to high humidity environments. Compared with previously reported ionic strain sensors [32, 33, 34, 35, 36], our interface‐modified piezoionic sensor exhibits superior comprehensive performance, characterized by relatively fast response and recovery time, high conductivity, excellent linearity, and good durability (Figure 3k). Table S2 compares the voltage signal of this sensor with those of previously reported piezoionic strain sensors, demonstrating its enhanced signal performance. These advantages clearly highlight the effectiveness of the interfacial modification strategy in enhancing piezoionic sensing performance, laying a solid foundation for high‐precision applications such as accurate motion monitoring and intelligent human‐machine interaction.

### 2.3 Practical Applications of Monitoring Human Motions

**FIGURE 4:**
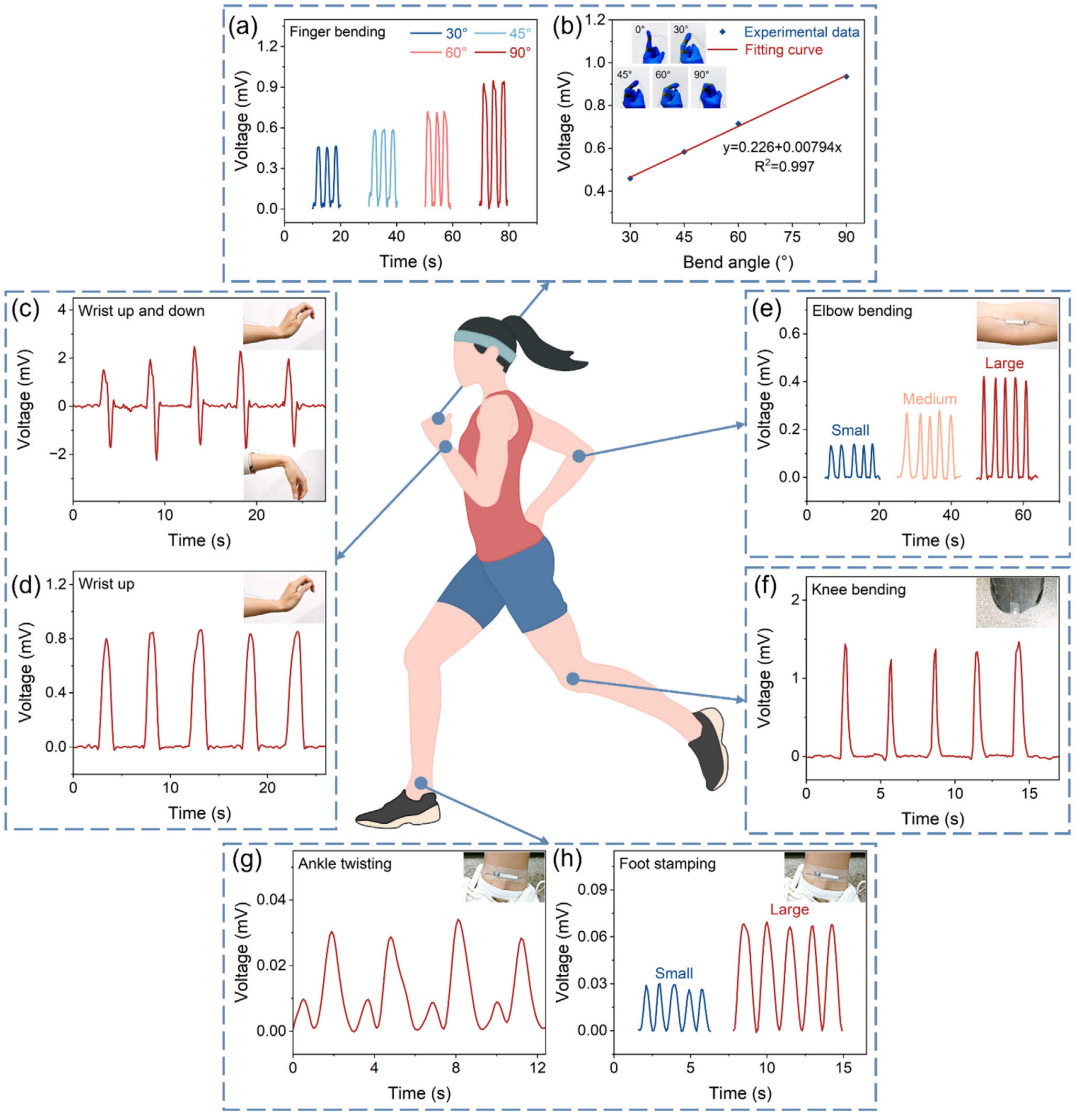
Piezoionic sensors based on BPNA/IL/BPNA composite film for real‐time human motion detection. (a) Voltage output signals of finger bending at different angles (30°, 45°, 60°, and 90°). (b) Experimental data along with the corresponding fitting curve. Voltage output signals of dynamic joint movements including: (c) wrist bending in upward and downward directions, (d) repeated upward wrist bending with consistent amplitude, (e) elbow bending, (f) knee bending, (g) ankle twisting, and (h) foot stamping.

Owing to its excellent sensitivity, flexibility, and rapid response time, the piezoionic sensor based on the BPNA/IL/BPNA composite film enables non‐invasive monitoring of various human physical and physiological parameters. When utilized for real‐time human motion monitoring, the piezoionic sensors can seamlessly fit joints (such as finger joints, wrist joints, ankle joints, and knee joints) using medical tape, as shown in Figure 4. The bending and releasing at joints induce shear deformation in the piezoionic sensor, resulting in voltage output signals. Detailed human motion information can be obtained based on the peak values, waveforms, and time intervals. When moving joints at the same amplitude, the voltage waveforms exhibit similar characteristics repeatedly, showing excellent repeatability and stability of the piezoionic sensor. It is worth noting that different bending amplitudes can also be accurately reflected in signal output. Connect the piezoionic sensor to a finger and detect bending signals in real‐time at different angles (30°, 45°, 60°, and 90°), as shown in Figure 4a,b. A linear relationship exists between the bending angle and the output voltage. The piezoionic sensor can differentiate the direction of human motion. When the wrist bends upward or downward, two opposing voltage signals are generated (Figure 4c). The sensor is placed on the back of the hand in Figure S20, and the states of fist clenching and relaxation can be identified from the acquired signals. Whether the movement is individual or continuous, and the amplitude of the movement is large or small, the piezoionic sensor can precisely respond to arm motions (Figure 4d,e; Figure S21). Furthermore, the strain sensor also enables real‐time monitoring of other joint movements, including knee (Figure 4f), ankle (Figure 4g,h), and neck (Figure S22). Although the ankle joint undergoes relatively subtle deformation during motion, our sensor still produces a reliable response with clear differentiation between ankle rotation and foot stamping. As discussed above, the sensor can maintain conformal contact with irregular human skin, withstand complex deformations, and enable clear, stable, and accurate monitoring of various body movements. The performance highlights its potential for high‐precision flexible sensing applications, including human posture monitoring and the prevention of sports injuries.

### 2.4 Practical Applications of Eye Tracking

**FIGURE 5:**
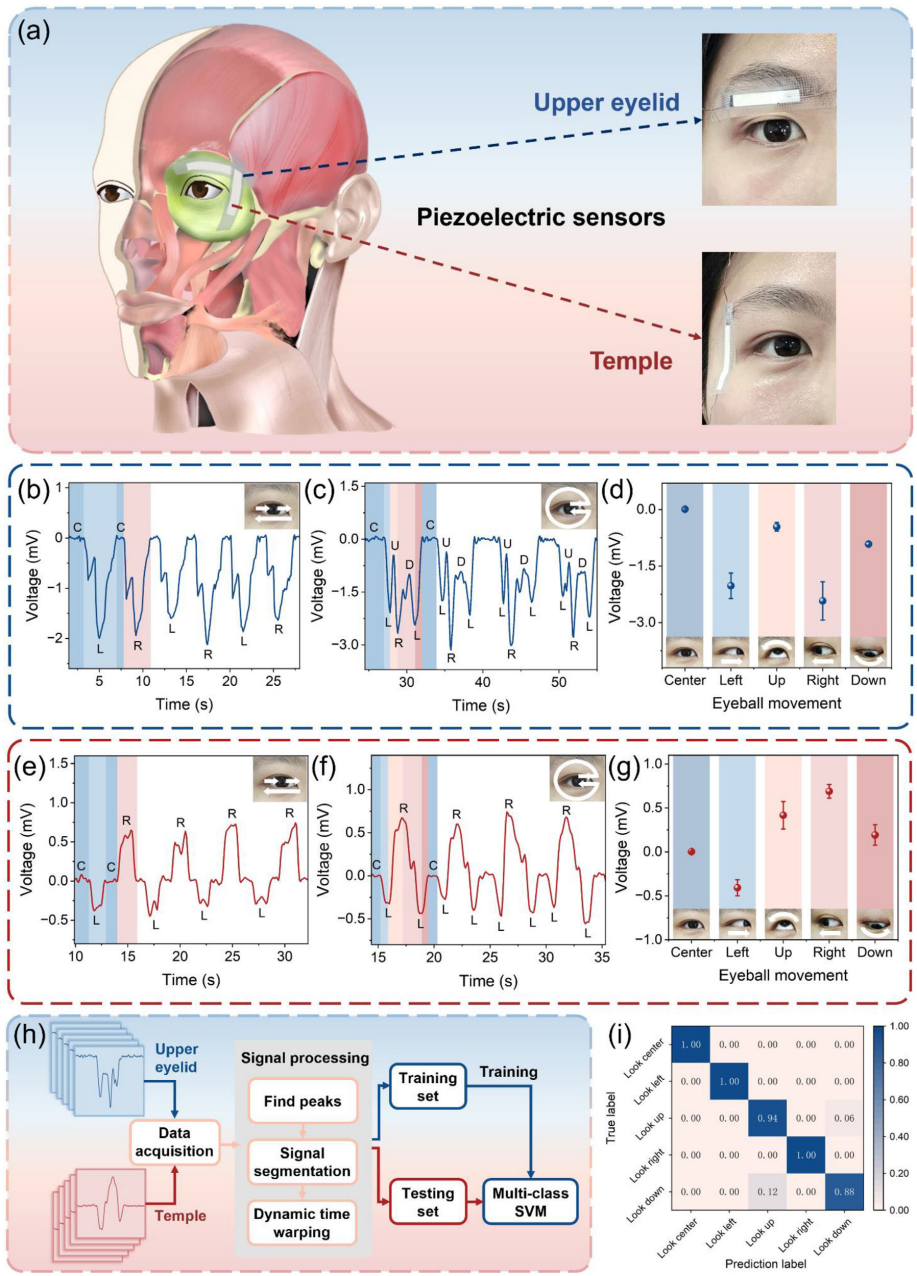
Piezoionic sensors based on BPNA/IL/BPNA composite film for eye tracking and machine learning. (a) Schematic illustration of the working principle for oculomotor monitoring. The sensors are respectively attached to the upper eyelid (the upper inset) and the temple (the lower inset). Voltage output signals from the U‐sensor of moving (b) laterally and (c) rotationally. Voltage output signals from the T‐sensor of moving (e) laterally and (f) rotationally. Comparison of average output signals of eyeball positions from (d) U‐sensor and (g) T‐sensor. (h) Flow chart of a machine learning algorithm for eyeball position classification. (i) Confusion matrix of classification training recognition accuracy.

In addition to detecting large‐scale deformations at joints, the flexible piezoionic sensor is also capable of detecting weak, slight, and local strains on the skin. As depicted in Figure 5, sensors are securely affixed with medical tape on the upper eyelid (U‐sensor) and temple (T‐sensor) of the right eye, enabling the monitoring of eye movement trajectory. Medical‐grade polyethylene tape could maintain strong adhesion while minimizing the risk of skin irritation or damage. Additionally, the sensor presents no toxicological hazard under intended use conditions. The electrode material carbon nanotubes have no direct contact with the human body, eliminating the risk of direct vascular exposure or inhalation [37]. The ionic liquid used has low volatility and is well confined within the electrolyte. During the testing process, the eyeballs of volunteers undergo slow and periodic lateral or rotational movements. Figure 5a illustrates the working principle: the sensors initially conform to the contour of the skin. Upon further bending caused by muscle contraction, tensile strain occurs, leading to a positive potential output. Conversely, muscle relaxation results in the sensor flattening, compressive strain occurs, and a negative potential output is measured. Since eye movements involve distinct muscle activities, sensors have the potential to distinguish different eye movements. The T‐sensor corresponds to the lateral rectus and facilitates horizontal movement detection, whereas the U‐sensor is associated with the superior rectus and superior oblique, enabling vertical and rotational movement detection [38, 39].

Figure 5b,c, respectively depict signal output characteristics of the U‐sensor when the eyeball moves laterally and rotationally. When the eyeball is initially in the center, the U‐sensor is under tension. As the eyeball migrates to other positions, the U‐sensor is released, and then negative signals are produced. Due to the larger deformation during lateral movement, the voltage signal amplitudes of the left (|2.02 mV|) and right (|2.43 mV|) positions are greater. Similarly, the voltage signal amplitude of the down position (|0.92 mV|) is slightly larger than that of the up position (|0.45 mV|). As shown in Figure 5e,f, the T‐sensor can effectively distinguish the direction of lateral movement. When the eyeball moves toward the temple side (right), the lateral rectus contracts, generating a positive voltage output. In contrast, when the eyeball shifts toward the nasal side (left), the lateral rectus relaxes, generating a negative voltage output. Figure 5d,g illustrate the average voltage output signals of eye movement position. By integrating the signals from the two sensors, distinguishable voltage signals can be obtained for the eyeball's position, thereby achieving eye tracking. Regions of distinct background colors in Figure 5c,f correspond to different phases of rotational movement, highlighting the potential to conduct intuitive and precise analysis of every step on continuous eye movement. Under ideal conditions, the voltage signals of the left and right positions from the U‐sensor would exhibit approximate numerical values. Similarly, the signals of the up and down positions by the T‐sensor would also show a similar consistency. Nonetheless, it is necessary to consider individual differences, such as variations in eye morphology and the orientation of periorbital muscles, and the subtle differences in sensor positions. These factors can lead to deviations in the above values.

To achieve human‐machine interaction applications, Support Vector Machine (SVM) is used as the supervised learning method (Figure 5h). We test 160 cycles of continuous eye movement signals, randomly selecting 60 of them as the training set and the remaining as the testing set. When processing signals, a dynamic threshold is set to detect effective signal segments. Notably, the particularity of T‐sensor lies in that its signal does not show obvious peaks at the up and down positions. Therefore, interpolation estimation based on the intermediate time points of adjacent left and right positions is required to supplement the data. A clustering strategy based on a dual threshold decision is employed for signal segmentation. Dynamic Time Warping (DTW) algorithm is used to align asynchronous periodic signals by establishing the mapping relationship between the periods of U‐sensor and T‐sensor. As shown in Figure 5i, the trained model can accurately distinguish five types of eye movement positions with a classification accuracy of 96.4%. Precise eye‐tracking technology can be widely applied in various fields [40, 41], including diagnosis and treatment of brain‐related diseases, cognitive function assessment, and driver fatigue detection. To facilitate natural and efficient human‐machine interaction, the convenience and comfort of wearable eye‐tracking devices are equally important. Most traditional devices, primarily depending on electrooculography (EOG) and video oculography (VOG) eye video analysis, require costly and bulky components [42]. Their tracking accuracy can be significantly compromised by environmental factors, thereby restricting their applicability in everyday consumer settings. In contrast, our sensors combine the advantages of high precision, miniaturization, and wearing comfort, expanding potential applications in healthcare, human‐machine interaction, and virtual reality.

### 2.5 Practical Applications of Respiratory and Swallowing Monitoring

**FIGURE 6:**
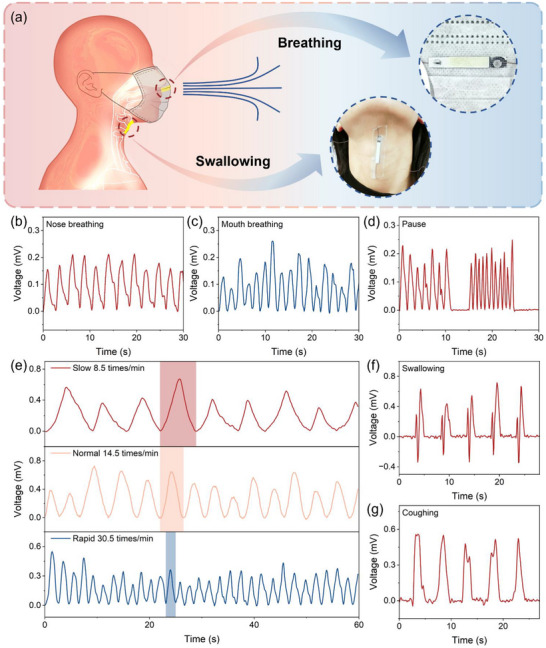
Piezoionic sensors based on BPNA/IL/BPNA composite film for respiratory and swallowing monitoring. (a) Schematic illustration of the working principle for respiratory and swallowing monitoring. The sensors are respectively attached to a commercial face mask (the upper inset) and the throat region (the lower inset). Respiratory monitoring with Tester 1 of (b) nose breathing, (c) mouth breathing, and (d) continuous breathing with breath‐holding. (e) Respiratory monitoring with Tester 2 of varying nose breathing rates. Voltage output signals from the throat region of (f) swallowing and (g) coughing.

Our sensor is capable of not only detecting the variations in force under direct contact but also measuring pressure in non‐contact scenarios, such as the air pressure generated by respiration. This feature demonstrates its excellent sensing performance under conditions of low amplitude and low frequency. The piezoionic sensor is attached to a commercial face mask, and the curved surface of the mask has no effect on the subsequent breathing test. Figure S23 presents the voltage response under static bending, the voltage signal decays and approaches zero under static strain. This behavior originates from the gradual decay of piezoelectric charges due to the non‐zero conductivity of the materials [43, 44]. Upon volunteers wearing the mask, the sensor is precisely aligned with the exhaled airflow from nose and has no direct contact with wearers (Figure 6a). The piezoionic sensor generates corresponding signal responses to dynamic breathing behaviors based on intensity and frequency of breathing. Figure 6b,c, respectively show the signal response curves of slow nose breathing and mouth breathing. And the voltage output signals of rapid breathing are displayed in Figure S24. Significant differences can be observed in both amplitude and period of the voltage signals, whereas the repeated sensing signals maintain nearly identical shapes. When Tester 1 holds the breath, an almost flat curve is observed. Figure 6d also demonstrates that the sensor can seamlessly respond to transitions among different respiratory states. Figure 6e shows the voltage output signals of Tester 2 under varying breathing rates. Respiratory monitoring curve of Tester 3 is provided in Figure S25. Distinct breathing signal curves can be observed among different testers, influenced by factors such as gender, body type, and age. The pattern and frequency of breathing contain rich physiological information related to human health. These signals can be utilized for detecting respiratory diseases such as sleep apnea, pneumonia, and asthma, as well as predicting acute events like cardiac arrest or sudden illnesses during physical activity [45, 46]. Therefore, the piezoionic sensor can acquire fundamental breathing information in a non‐invasive and non‐contact way, including breathing patterns, breathing habits, and breathing disorders, thereby achieving simple, real‐time, and straightforward monitoring and assessment of human health.

Meanwhile, we also conformally attach the sensor to the neck, corresponding to the laryngeal prominence, to detect laryngeal cartilage and muscle movements during swallowing and coughing events (Figure 6a). As shown in Figure 6f, the piezoionic sensor exhibits high sensitivity to swallowing movements, and the output voltage signals can effectively capture the two distinct phases of the swallowing process [47]. The first signal peak occurs when the laryngeal cartilage moves upward, passing and pushing against the sensors. Conversely, the secondary signal peak corresponds to the downward movement of the laryngeal cartilage back to its initial position, again passing and pushing against the sensors. From this, two important parameters of swallowing can be obtained: swallowing frequency and duration. These parameters serve as key indicators for assessing dysphagia and associated complications, enabling an objective evaluation of swallowing ability [48]. Currently, one of the commonly used swallowing assessment methods is videofluoroscopy, which necessitates costly equipment and is restricted in usage duration owing to radiation exposure concerns [49]. Another approach, acoustic monitoring, is limited to quiet environments due to its sensitivity to body noise and external noise, requiring more sophisticated algorithms to mitigate interference. In contrast, the developed sensor offers a safe and effective solution for long‐term swallowing monitoring and evaluation. When integrated with respiratory monitoring sensor signals, it allows for the investigation of the interaction between breathing and swallowing. The rhythmic coordination between breathing and swallowing is crucial for normal pharyngeal function. This technology holds significant promise for daily monitoring and therapeutic interventions in patients with cardiopulmonary comorbidity and upper respiratory tract disorders [50, 51]. In addition, the piezoionic sensor can detect the signal of coughing, clearly distinguishing it from swallowing (Figure 6g). When mutually confirmed with respiratory monitoring signals, it can more accurately check for diseases such as chronic bronchitis and asthma [47].

## 3 Conclusion

In summary, by employing a piezoelectric interface modification strategy, we successfully amplify the piezoionic effect and fabricate a high‐performance piezoionic sensor. We discover that the built‐in electric field based on piezoelectric interfaces generates a driving force for ionic transport, significantly accelerating ionic migration and promoting the accumulation of polarized ions at the interface. Due to the regulation of ionic dynamics, our sensor exhibits excellent sensing performance, with a conductivity of 14.4 µS/cm, a fast response time of 40 ms, and a long‐term cycling stability up to 4000 times. In practical applications, our sensor conforms well to human skin, enabling real‐time detection of large‐scale at the joint and slight local strains on the skin with high sensitivity. This work provides a new general principle and structural design for improving the performance of flexible piezoionic sensors, and paves the way for their broader application in future wearable and biomedical technologies.

## 4 Materials and Methods

### 4.1 Materials

N,N'‐methylenebis(4‐nitroaniline) (BPNA) was purchased from Sigma Aldrich, N,N‐dimethylformamide (DMF) and ethanol were purchased from Chinasun Specialty Products Co., Ltd, 1‐ethyl‐3‐methylimidazolium bis(trifluoromethylsulfonyl)imide (EMIMTFSI) was purchased from Shanghai Cheng Jie Chemical Company, poly(ethylene oxide) (PEO) was purchased from Hefei BASF Biotechnology Co., Ltd, multi‐walled carbon nanotubes (MWCNTs) were purchased from Xianfeng Nano Company, and N‐methylpyrrolidone (NMP) was purchased from Sinopharm Chemical Regent Co. Ltd. All the reagents were analytically pure and used as received.

### 4.2 Preparation of BPNA Crystals

50 mg of BPNA powder was dissolved in 1 mL of DMF in a clean sample bottle, and then 3.5 mL of ethanol was slowly added to the upper layer of the solution. The resulting mixture was maintained at room temperature for 1–2 days to allow slow diffusion, and yellow needle‐shaped BPNA crystals were obtained. The mixture was filtered and washed several times to remove the residual solvent, and finally dry at 60°C to obtain grown BPNA crystals.

### 4.3 Preparation of the BPNA/IL/BPNA Composite Film

First, 1 g of PEO was dissolved into 10 mL of deionized water under continuous stirring at room temperature for 5 h. To prepare the PEO/BPNA composite film, grown BPNA crystals were added to the PEO solution with different weight percentages (10‐30 wt.%). The resulting mixture was then poured onto a glass template and dried at 60 °C for 1 h. Further to prepare the PEO/IL film, 0.5 g of EMIMTFSI was dispersed into 10 mL of PEO solution under continuous stirring for 3 h. The fully dispersed mixed solution was poured onto the PEO/BPNA film and dried at 60°C for 2 h to form the PEO/IL intermediate layer. By repeating the above steps, another layer of PEO/BPNA was poured onto the PEO/IL layer and dried, resulting in the composite film with PEO/BPNA interface modification.

### 4.4 Fabrication of Piezoionic Sensors Based on the BPNA/IL/BPNA Composite Film

First, 20 mg of MWCNTs were dispersed in 20 mL of NMP via ultrasonic treatment for 40 min to form a homogeneous suspension. Subsequently, 3 mL of the resulting suspension was vacuum‐filtered and dried at 120°C for 5 h to obtain the MWCNTs films. The prepared ionogel films and MWCNTs films were cut into rectangular shapes measuring 2 cm × 0.5 cm and 3 cm × 0.5 cm, respectively. Two MWCNT films were attached to both sides of the ionogel film as top and bottom electrodes in the device. The flexible piezoionic sensor was then assembled through hot pressing at 70°C for 3 h. The PEO/BPNA piezoelectric and PEO/IL piezoionic sensors were fabricated using the same procedure as described above.

### 4.5 Characterizations

The morphology and structure of the crystal and sensor were obtained by scanning electron microscope (Hitachi SU8010) and hot‐stage polarizing light microscope (Leica DMRX). XRD patterns were obtained on X'Pert‐Pro MPD (Cu‐Ka). Mechanical properties were tested using a Zwick Roell Z0.5 material testing machine.

Electrochemical properties of the sensor were carried out with a CHI660E electrochemical working station. For the bending sensing detection, the sensor was attached to the surface of a flexible substrate and suspended between the movable platform with two ends fixed. A stepper motor was used to drive one stage in uniaxial motion while the other remained stationary, enabling controlled tensile‐compressive bending of the sensor. The bending axis was aligned with the geometric center of the electrolyte layer to ensure uniform strain distribution across the active region. At the start of each test, the initial center‐to‐center distance between the stages was set to 50 mm. For the measurement, no voltage was applied on the sensor. In the bending tests not otherwise specified, the displacement was set at 5 mm.

All electrochemical impedance spectroscopy measurements were performed on a CHI660E electrochemical working station and analyzed with a Metrohm Autolab NOVA Electrochemical Software. The applied AC potential was 10 mV and the frequency scanned from 0.01 Hz to 1 MHz. All measurements were conducted under standard laboratory conditions at 25°C ± 2°C and 50% ± 10% relative humidity. Impedance was scanned several times until it became stabilized after the temperature was changed.

## Author Contributions

Y.Y.C., X.H.Z., and C.L. conceived and designed the experiments. Y.Y.C. and R.Z.G. performed most experiments. Y.Y.C., X.Y.L., and R.Z.G. analyzed data. Y.Y.C. and C.L. wrote and checked the manuscript. X.C. and C.L. supervised the project.

## Ethical Approval Statement

This study was conducted after obtaining ethical approval granted by the Ethics Committee of Soochow University (Approval Project No. SUDA20250327H13).

## Conflicts of Interest

The authors declare no conflicts of interest.

## Supporting information




**Supporting File 1**: advs74757‐sup‐0001‐SuppMat.docx.

**Supporting File 2**: advs74757‐sup‐0002‐MovieS1.mp4.

**Supporting File 3**: advs74757‐sup‐0003‐MovieS2.mp4.

## Data Availability

The data that support the findings of this study are available from the corresponding author upon reasonable request.

## References

[1] A. Waseem , A. Abdullah , I. V. Bagal , J.‐S. Ha , J. K. Lee , and S.‐W. Ryu , “Self‐Powered and Flexible Piezo‐Sensors Based on Conductivity‐Controlled GaN Nanowire‐Arrays for Mimicking Rapid‐ and Slow‐Adapting Mechanoreceptors,” npj Flexible Electronics 6 (2022): 58, 10.1038/s41528-022-00197-1.

[2] Y. Y. Chen , X. H. Zhang , and C. Lu , “Flexible Piezoelectric Materials and Strain Sensors for Wearable Electronics and Artificial Intelligence Applications,” Chemical Science 15 (2024): 16436–16466, 10.1039/D4SC05166A. 39355228 PMC11440360

[3] S. Min , D. H. Kim , D. J. Joe , et al., “Clinical Validation of a Wearable Piezoelectric Blood‐Pressure Sensor for Continuous Health Monitoring,” Advanced Materials 35 (2023): 2301627, 10.1002/adma.202301627. 36960816

[4] Z. Yi , Z. Liu , W. Li , et al., “Piezoelectric Dynamics of Arterial Pulse for Wearable Continuous Blood Pressure Monitoring,” Advanced Materials 34 (2022): 2110291, 10.1002/adma.202110291. 35285098

[5] H.‐S. Wu , S.‐M. Wei , S.‐W. Chen , et al., “Metal‐Free Perovskite Piezoelectric Nanogenerators for Human–Machine Interfaces and Self‐Powered Electrical Stimulation Applications,” Advanced Science 9 (2022): 2105974, 10.1002/advs.202105974. 35445556 PMC9218782

[6] Y.‐X. Zhou , Y.‐T. Lin , S.‐M. Huang , et al., “Tungsten Disulfide Nanosheets for Piezoelectric Nanogenerator and Human‐Machine Interface Applications,” Nano Energy 97 (2022): 107172, 10.1016/j.nanoen.2022.107172.

[7] Y. Dobashi , D. Yao , Y. Petel , et al., “Piezoionic Mechanoreceptors: Force‐Induced Current Generation in Hydrogels,” Science 376 (2022): 502–507, 10.1126/science.aaw1974. 35482868

[8] Y. X. Chen , Y. Y. Chen , R. Z. Gao , X. P. Yu , and C. Lu , “Reversible Molecule Interactions Enable Ultrastretchable and Recyclable Ionogels for Wearable Piezoionic Sensors,” ACS Applied Materials & Interfaces 16 (2024): 50027–50035, 10.1021/acsami.4c11268. 39270305

[9] Y. Sun , G. Tian , T. Yang , et al., “Crosslinking‐Modulated Hydrogel Piezoionic Sensor for Pattern Security Authentication in Human‐Machine Interfaces,” Advanced Functional Materials 35 (2025): 2420187, 10.1002/adfm.202420187.

[10] D. Ho , “The Piezoionic Effect: Biomimetic Transduction Mechanism for Sensing, Actuation, Interface, and Energy Harvesting,” ChemElectroChem 11 (2023): 202300268, 10.1002/celc.202300268.

[11] J. G. Xu , Q. Li , and D. Ho , “A Universal Framework for Determining the Effect of Operating Parameters on Piezoionic Voltage Generation,” Materials Horizons 11 (2024): 5709–5721, 10.1039/D4MH01067A. 39234925

[12] Y. Deng , J. Liu , Y. T. Lei , G. Y. Huang , Z. Zhang , and J. L. Sha , “Anisotropic Cellulose Nanofibril Piezoionic Organohydrogel Fabricated by Directional Freezing for Flexible Strain Sensors,” International Journal of Biological Macromolecules 307 (2025): 142187, 10.1016/j.ijbiomac.2025.142187. 40101834

[13] X. Lu , Y. Chen , Y. Zhang , et al., “Piezoionic High Performance Hydrogel Generator and Active Protein Absorber via Microscopic Porosity and Phase Blending,” Advanced Materials 36 (2024): 2307875, 10.1002/adma.202307875. 37983590

[14] C. Lu and X. H. Zhang , “Accurate Blood Pressure Detection Enabled by Graphdiyne Piezoionic Sensors With Ultrafast out‐Plane Ion Transfer,” Carbon 222 (2024): 118956, 10.1016/j.carbon.2024.118956.

[15] D. Lv , X. Li , X. Huang , et al., “Microphase‐Separated Elastic and Ultrastretchable Ionogel for Reliable Ionic Skin With Multimodal Sensation,” Advanced Materials 36 (2024): 2309821, 10.1002/adma.202309821. 37993105

[16] K. Yang , B. L. Li , Z. H. Ma , et al., “Ion‐Selective Mobility Differential Amplifier: Enhancing Pressure‐Induced Voltage Response in Hydrogels,” Angewandte Chemie International Edition 64 (2025): 202415000, 10.1002/anie.202415000. 39545315

[17] Y. X. Chen , Y. Y. Chen , X. Y. Ling , J. Y. Nie , and C. Lu , “Robust Ionogels With Slide‐Ring Structure for Wearable Piezoionic Sensors,” ACS Applied Polymer Materials 7 (2025): 10981–10986, 10.1021/acsapm.5c02531.

[18] X. Guan , S. Zheng , J. Luo , et al., “Spider Webs‐Inspired Aluminum Coordination Hydrogel Piezoionic Sensors for Tactile Nerve Systems,” Advanced Functional Materials 35 (2025): 2414016, 10.1002/adfm.202414016.

[19] Z. Yang , Q. Duan , J. Zang , et al., “Boron Nitride‐Enabled Printing of a Highly Sensitive and Flexible Iontronic Pressure Sensing System for Spatial Mapping,” Microsystems & Nanoengineering 9 (2023): 68, 10.1038/s41378-023-00543-x. 37251710 PMC10220000

[20] V. Amoli , J. S. Kim , E. Jee , et al., “A Bioinspired Hydrogen Bond‐Triggered Ultrasensitive Ionic Mechanoreceptor Skin,” Nature Communications 10 (2019): 4019, 10.1038/s41467-019-11973-5. PMC6728325 31488820

[21] Y.‐R. Kim , G. Lim , H. Cho , et al., “Bilayer Piezoionic Sensors for Enhanced Detection of Dynamic, Static, and Directional Forces With Self‐Healing Capabilities,” Nano Energy 127 (2024): 109749, 10.1016/j.nanoen.2024.109749.

[22] F. Yang , J. Li , Y. Long , et al., “Wafer‐Scale Heterostructured Piezoelectric Bio‐Organic Thin Films,” Science 373 (2021): 337–342, 10.1126/science.abf2155. 34437153 PMC8516594

[23] S. Bhunia , S. K. Karan , R. Chowdhury , et al., “Mechanically Flexible Piezoelectric Organic Single Crystals for Electrical Energy Harvesting,” Chemistry 10 (2024): 1741–1754, 10.1016/j.chempr.2024.01.019.

[24] C. B. Zhu , H. Cheng , and Y. Yang , “Electrochemical Characterization of Two Types of PEO‐Based Polymer Electrolytes With Room‐Temperature Ionic Liquids,” Journal of The Electrochemical Society 155 (2008): A569–A575, 10.1149/1.2931523.

[25] Q. Yu , Y. Bai , Z. Li , et al., “Interface‐Induced High Piezoelectric γ‐Glycine‐Based Flexible Biodegradable Films,” Nano Energy 121 (2024): 109196, 10.1016/j.nanoen.2023.109196.

[26] I. You , D. G. Mackanic , N. Matsuhisa , et al., “Artificial Multimodal Receptors Based on Ion Relaxation Dynamics,” Science 370 (2020): 961–965, 10.1126/science.aba5132. 33214277

[27] R. A. Huggins , “Simple Method to Determine Electronic and Ionic Components of the Conductivity in Mixed Conductors a Review,” Ionics 8 (2002): 300–313, 10.1007/BF02376083.

[28] V. R. Feig , H. Tran , M. Lee , and Z. A. Bao , “Mechanically Tunable Conductive Interpenetrating Network Hydrogels That Mimic the Elastic Moduli of Biological Tissue,” Nature Communications 9 (2018): 2740, 10.1038/s41467-018-05222-4. PMC6048132 30013027

[29] Y. Liu , Y. Hu , J. Zhao , G. Wu , X. Tao , and W. Chen , “Self‐Powered Piezoionic Strain Sensor Toward the Monitoring of Human Activities,” Small 12 (2016): 5074–5080, 10.1002/smll.201600553. 27150115

[30] W. Zhao , T. Sun , Y. Zheng , et al., “Tailoring Intermolecular Interactions Towards High‐Performance Thermoelectric Ionogels at Low Humidity,” Advanced Science 9 (2022): 2201075, 10.1002/advs.202201075. 35478492 PMC9284173

[31] S. A. Hashmi , “Influence of Water Absorption on Poly–Ethylene Oxide‐Based Polymer Electrolytes Complexed With Ammonium, Sodium and Magnesium Perchlorates,” Journal of Materials Science 33 (1998): 989–993, 10.1023/A:1004315912716.

[32] X. P. Yu , X. H. Zhang , and C. Lu , “Skin‐Inspired and Self‐Powered Piezoionic Sensors for Smart Wearable Applications,” Small 21 (2025): 2410594, 10.1002/smll.202410594. 39780614

[33] A. Yin , J. Wang , S. Hu , et al., “High Performance Waterproof‐Breathable Fully Flexible Tactile Sensor Based on Piezotronics Coupled OFET,” Nano Energy 106 (2023): 108034, 10.1016/j.nanoen.2022.108034.

[34] D.‐S. Liu , H. Ryu , U. Khan , et al., “Piezoionic‐Powered Graphene Strain Sensor Based on Solid Polymer Electrolyte,” Nano Energy 81 (2021): 105610, 10.1016/j.nanoen.2020.105610.

[35] X. Y. Yin , Y. Zhang , X. B. Cai , Q. Q. Guo , J. Yang , and Z. L. Wang , “3D Printing of Ionic Conductors for High‐Sensitivity Wearable Sensors,” Materials Horizons 6 (2019): 767–780, 10.1039/C8MH01398E.

[36] E. K. Boahen , B. Pan , H. Kweon , et al., “Ultrafast, Autonomous Self‐Healable Iontronic Skin Exhibiting Piezo‐Ionic Dynamics,” Nature Communications 13 (2022): 7699, 10.1038/s41467-022-35434-8. PMC9744819 36509757

[37] Y. Cao and Y. Luo , “Pharmacological and Toxicological Aspects of Carbon Nanotubes (CNTs) to Vascular System: A Review,” Toxicology and Applied Pharmacology 385 (2019): 114801, 10.1016/j.taap.2019.114801. 31678607

[38] N.‐I. Kim , J. Chen , W. Wang , et al., “Skin‐Attached Arrayed Piezoelectric Sensors for Continuous and Safe Monitoring of Oculomotor Movements,” Advanced Healthcare Materials 13 (2024): 2303581, 10.1002/adhm.202303581. 38386698

[39] N. I. Kim , J. Chen , W. J. Wang , et al., “Highly‐Sensitive Skin‐Attachable Eye‐Movement Sensor Using Flexible Nonhazardous Piezoelectric Thin Film,” Advanced Functional Materials 31 (2021): 2008242, 10.1002/adfm.202008242.

[40] L. Zhu , J. Chen , H. Yang , et al., “Wearable Near‐Eye Tracking Technologies for Health: A Review,” Bioengineering 11 (2024): 738, 10.3390/bioengineering11070738. 39061820 PMC11273595

[41] G. Borghini , L. Astolfi , G. Vecchiato , D. Mattia , and F. Babiloni , “Measuring Neurophysiological Signals in Aircraft Pilots and Car Drivers for the Assessment of Mental Workload, Fatigue and Drowsiness,” Neuroscience & Biobehavioral Reviews 44 (2014): 58–75, 10.1016/j.neubiorev.2012.10.003. 23116991

[42] S. Ban , Y. J. Lee , K. R. Kim , J. H. Kim , and W. H. Yeo , “Advances in Materials, Sensors, and Integrated Systems for Monitoring Eye Movements,” Biosensors 12 (2022): 1039, 10.3390/bios12111039. 36421157 PMC9688058

[43] A. K. Ramanathan , L. M. Headings , and M. J. Dapino , “Near Static Strain Measurement With Piezoelectric Films,” Sensors and Actuators A: Physical 301 (2020): 111654, 10.1016/j.sna.2019.111654.

[44] M. Xue , Y. Tang , Z. Shan , et al., “Deciphering the Leakage Conduction Mechanism of BiFeO_3_–BaTiO_3_ Lead‐Free Piezoelectric Ceramics,” Journal of Advanced Ceramics 12 (2023): 1844–1856, 10.26599/JAC.2023.9220792.

[45] J. Kim , H. Roh , S. Moon , et al., “Wireless Breathable Face Mask Sensor for Spatiotemporal 2D Respiration Profiling and Respiratory Diagnosis,” Biomaterials 309 (2024): 122579, 10.1016/j.biomaterials.2024.122579. 38670033

[46] C. Lu , X. P. Yu , Y. X. Chen , X. Chen , and X. H. Zhang , “Giant Piezoionic Effect of Ultrathin MXene Nanosheets Toward Highly‐Sensitive Sleep Apnea Diagnosis,” Chemical Engineering Journal 463 (2023): 142523, 10.1016/j.cej.2023.142523.

[47] L. Natta , F. Guido , L. Algieri , et al., “Conformable AlN Piezoelectric Sensors as a Non‐Invasive Approach for Swallowing Disorder Assessment,” ACS Sensors 6 (2021): 1761–1769, 10.1021/acssensors.0c02339. 34010558 PMC8294609

[48] A. M. Namasivayam‐MacDonald , C. E. A. Barbon , and C. M. Steele , “A Review of Swallow Timing in the Elderly,” Physiology & Behavior 184 (2018): 12–26, 10.1016/j.physbeh.2017.10.023. 29101012 PMC5742298

[49] M. Golabbakhsh , A. Rajaei , M. Derakhshan , S. Sadri , M. Taheri , and P. Adibi , “Automated Acoustic Analysis in Detection of Spontaneous Swallows in Parkinson's Disease,” Dysphagia 29 (2014): 572–577, 10.1007/s00455-014-9547-4. 24958599

[50] I. H. Gewolb , D. Fishman , M. A. Qureshi , and F. L. Vice , “Coordination of Suck‐ Swallow‐Respiration in Infants Born to Mothers With Drug‐Abuse Problems,” Developmental Medicine & Child Neurology 46 (2004): 700–705, 10.1111/j.1469-8749.2004.tb00984.x. 15473175

[51] E. Reynolds , D. Grider , R. Caldwell , et al., “Swallow–Breath Interaction and Phase of Respiration With Swallow During Nonnutritive Suck Among Low‐Risk Preterm Infants,” American Journal of Perinatology 27 (2010): 831–840, 10.1055/s-0030-1262504. 20607645

