# Supplementary material for: Artificial Piezoelectric Interface Enables Ultrafast Interfacial Ion Kinetic for Highly‐Sensitive Piezoionic Sensors
Source: Adv Sci (Weinh). 2026 Mar 10;13(29):e74757. doi: 10.1002/advs.74757 (PMC13205602; doi:10.1002/advs.74757)
Supplement: Supplementary file 1 — Supporting File 1: advs74757‐sup‐0001‐SuppMat.docx. [file ADVS-13-e74757-s002.docx]

Supporting Information

**Artificial piezoelectric interface enables ultrafast interfacial ion kinetic for highly-sensitive piezoionic sensors**

Yanyu Chen^1^, Xingyue Ling^1^, Rizhong Gao^1^, Xiaohong Zhang^2^, Xi Chen^3^ and Chao Lu^1,*^

^1^ College of Chemistry, Chemical Engineering and Materials Science, Soochow University, Suzhou, Jiangsu 215123, China.

^2^ Institute of Functional Nano & Soft Materials, Soochow University, Suzhou, Jiangsu 215123, China.

^3^ Department of Earth and Environmental Engineering, Columbia University, New York, NY 10027, USA.

* Corresponding author. Email: chaolu@suda.edu.cn


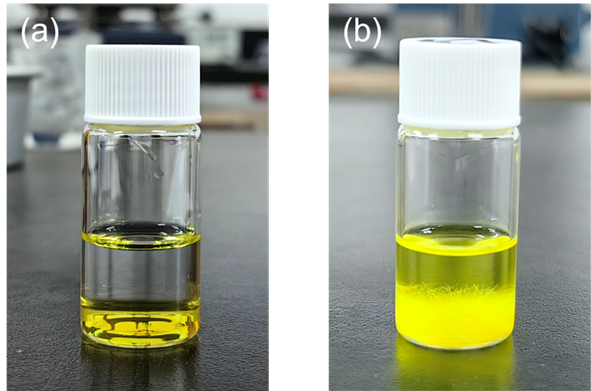


**Figure S1.** Optical images of (a) liquid-liquid diffusion method and (b) the cultivated yellow needle-shaped BPNA crystals.


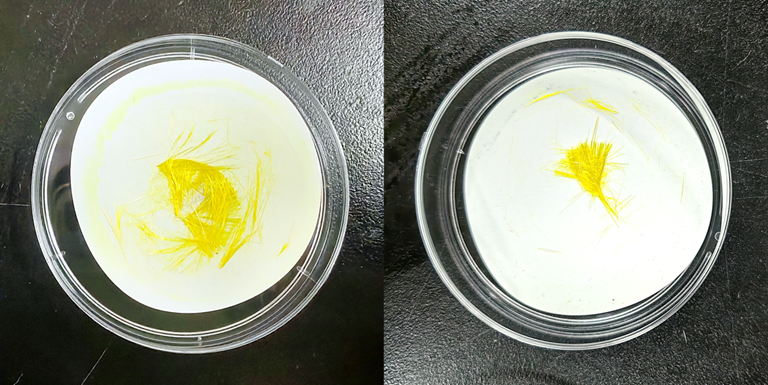


**Figure S2.** Optical images of BPNA crystals under bending states.


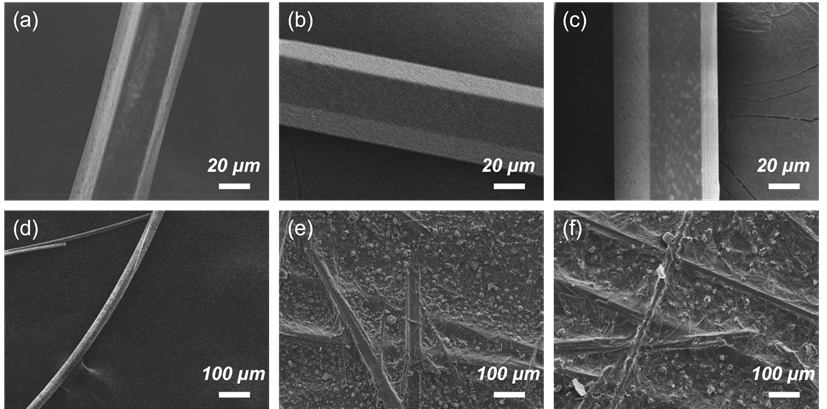


**Figure S3.** SEM images showing (a) (b) (c) hexagonal morphology of the BPNA crystals, (d) bent shape of BPNA needle crystals, and (e) (f) surface morphology of the BPNA/IL/BPNA composite film. The BPNA crystals in the composite film show hexagonal morphology.


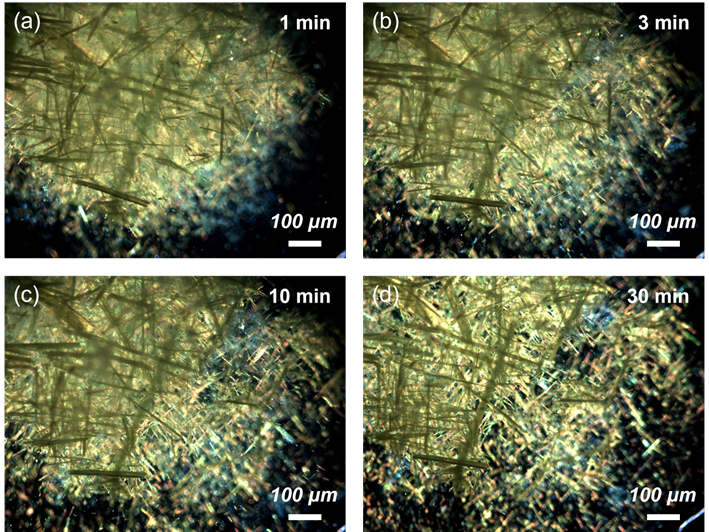


**Figure S4.** HSPM images of the BPNA/IL/BPNA composite film, showing degradation at room temperature in an aqueous solution within the initial 30 minutes.


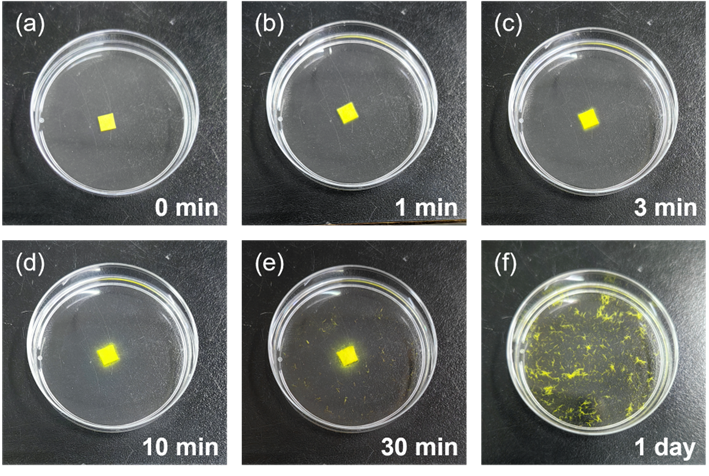


**Figure S5.** Degradation evaluation of the BPNA/IL/BPNA composite film in deionized water. The composite film dissolved in water within 1 day.


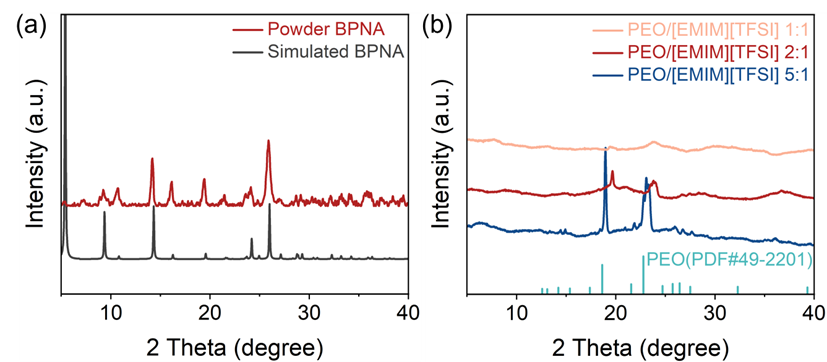


**Figure S6.** XRD characterization of (a) BPNA crystal powder and (b) PEO/IL films with different IL contents.


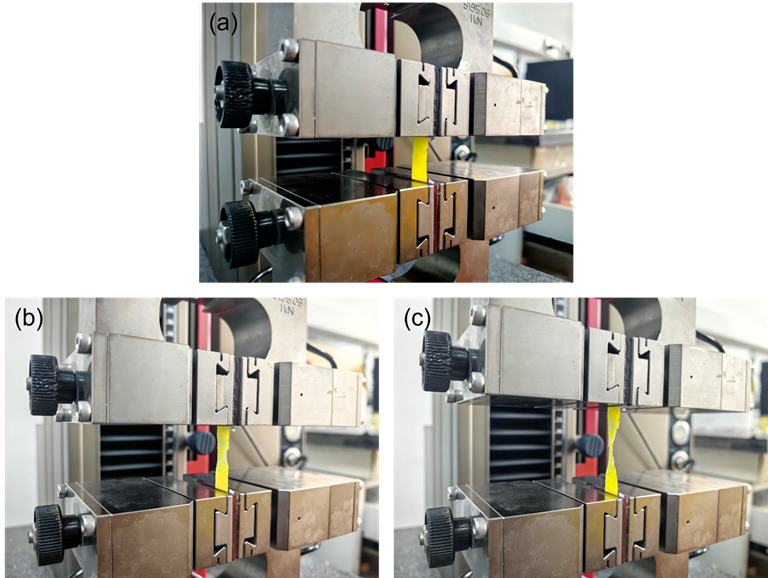


**Figure S7.** Optical images of the BPNA/IL/BPNA composite film under tensile testing. (a) Initial state. (b) Cracks appear at 59% strain. (c) Almost fractured at 100% strain.


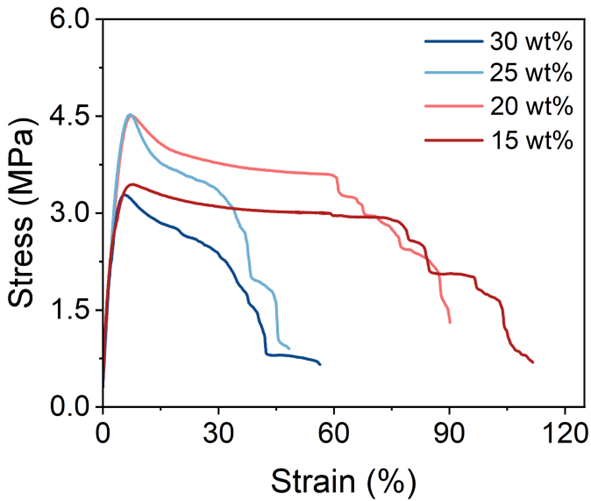


**Figure S8.** Strain-stress curves of the BPNA/IL/BPNA composite films with different BPNA crystal contents.


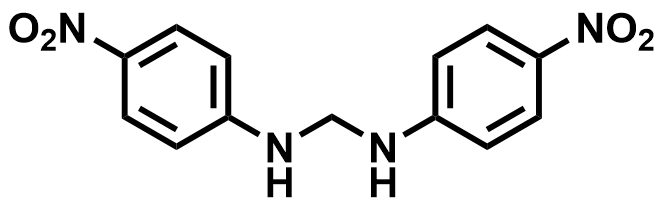


**Figure S9.** The chemical structural formula of BPNA.


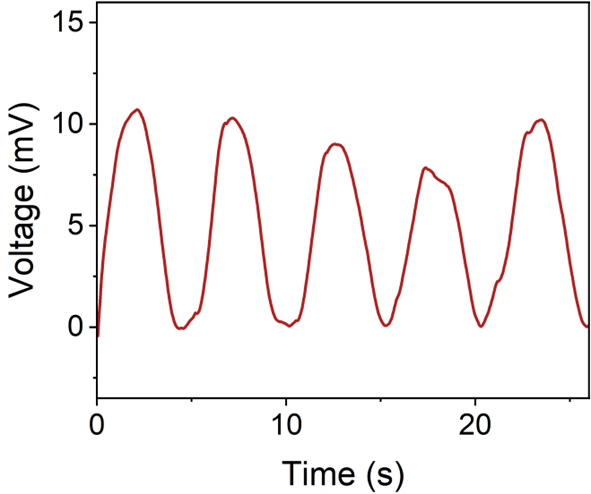


**Figure S10.** Voltage output signals of a needle-shaped BPNA crystal bending.


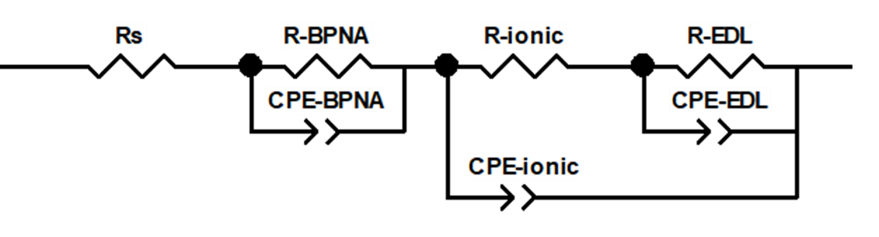


**Figure S11.** Equivalent circuit model representing the sensor based on BPNA/IL/BPNA composite film.


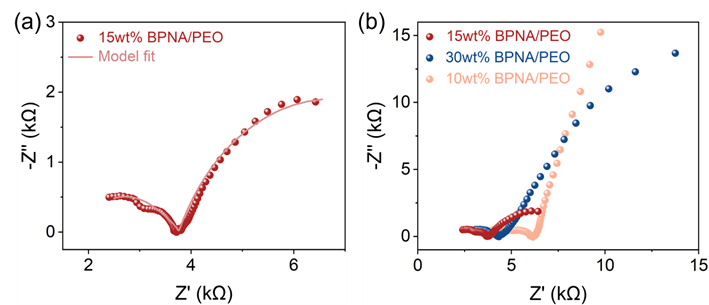


**Figure S12.** (a) Nyquist plot and fitted curve for the sensor with 15 wt% BPNA crystal content. (b) Comparison of Nyquist plots with different BPNA crystal contents.


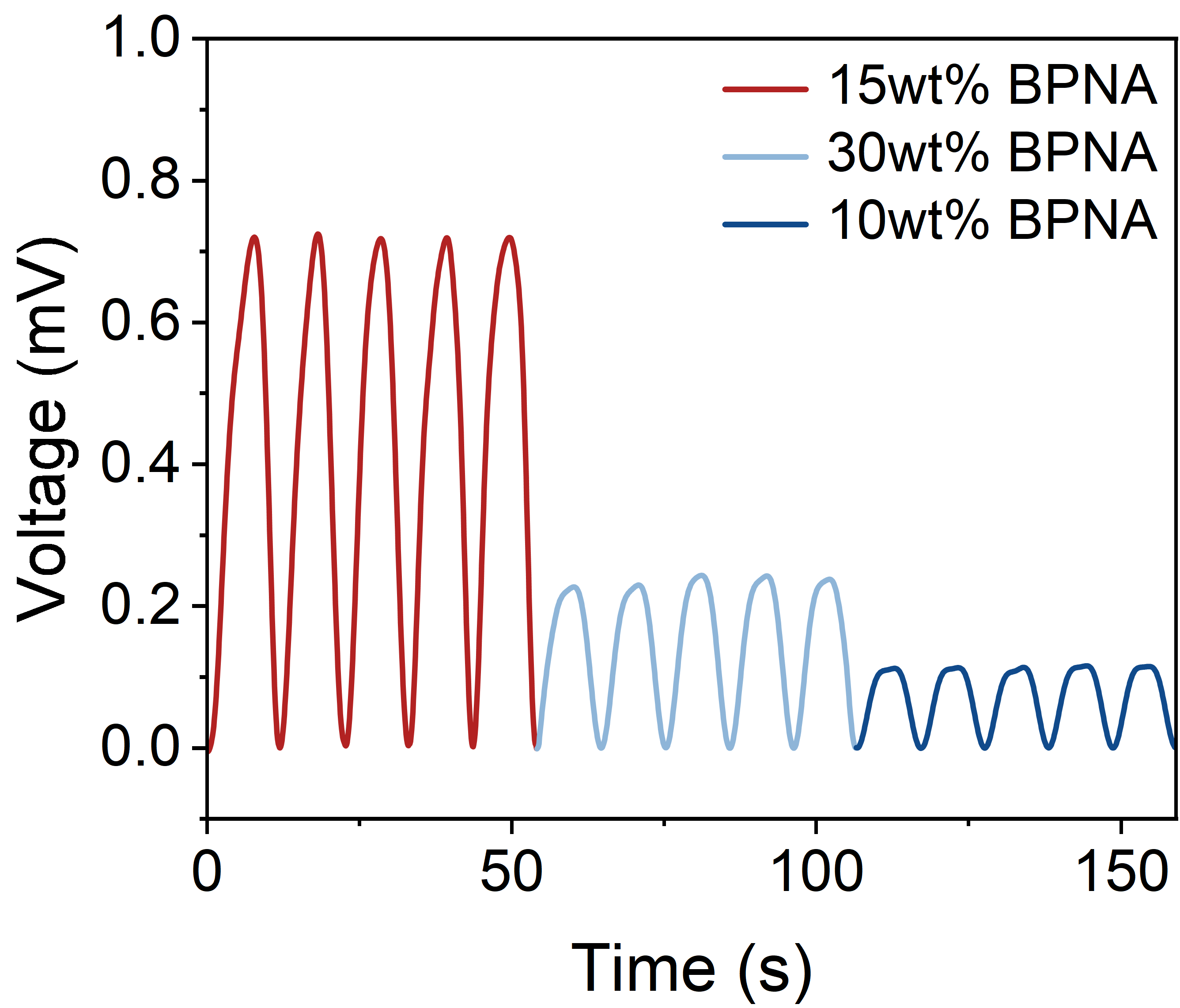


**Figure S13**. Comparison of voltage signals between the piezoelectric PEO/BPNA sensors with different BPNA crystal contents.


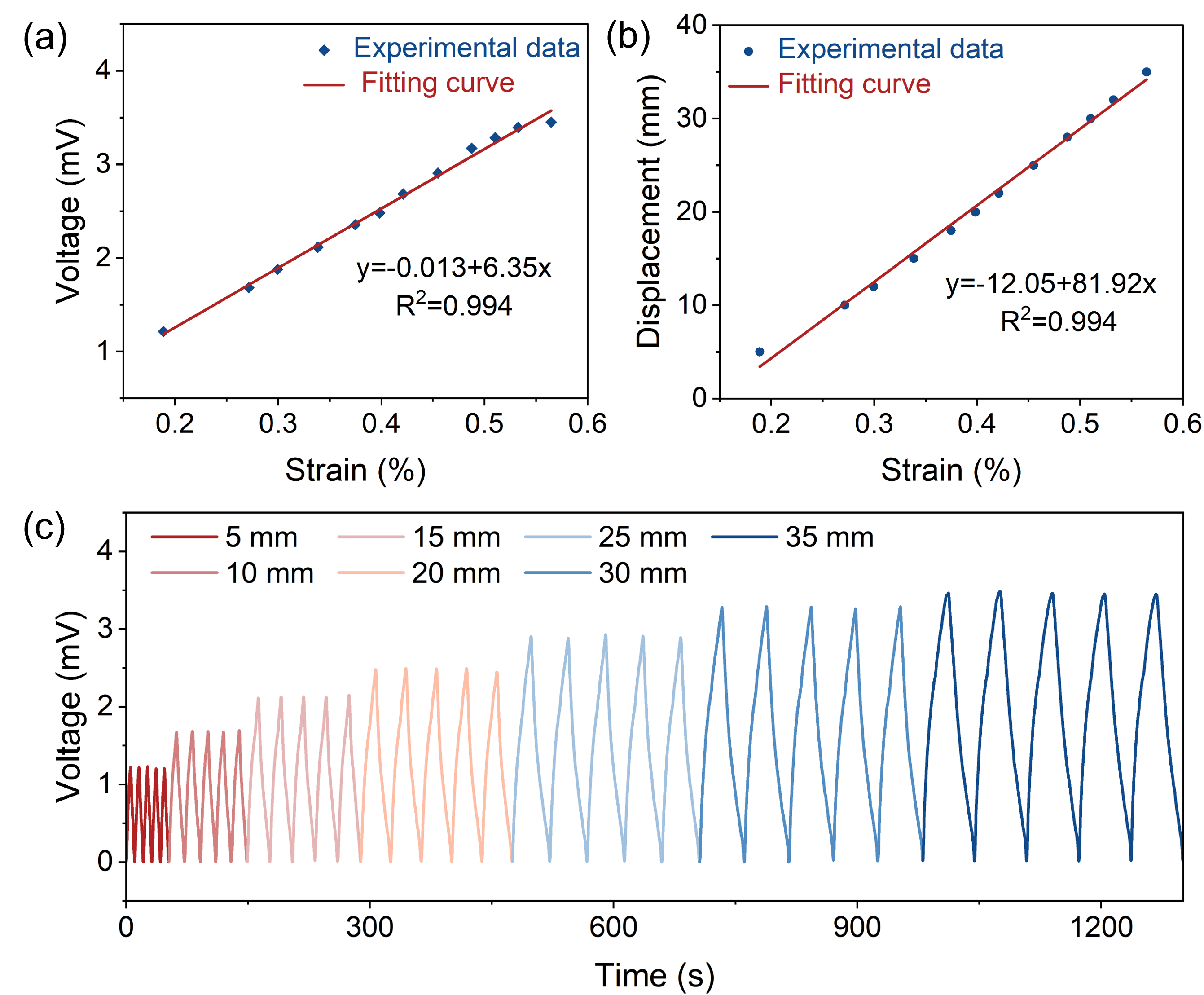


**Figure S14.** Experimental data along with the corresponding fitting curve for (a) voltage signals of the sensor under various bending strain, and (b) bending strain at varying displacements. (c) Sensing response of the sensor based on BPNA/IL/BPNA composite film under elastic bending deformations with displacements ranging from 5 mm (0.19% strain) to 35 mm (0.56% strain).


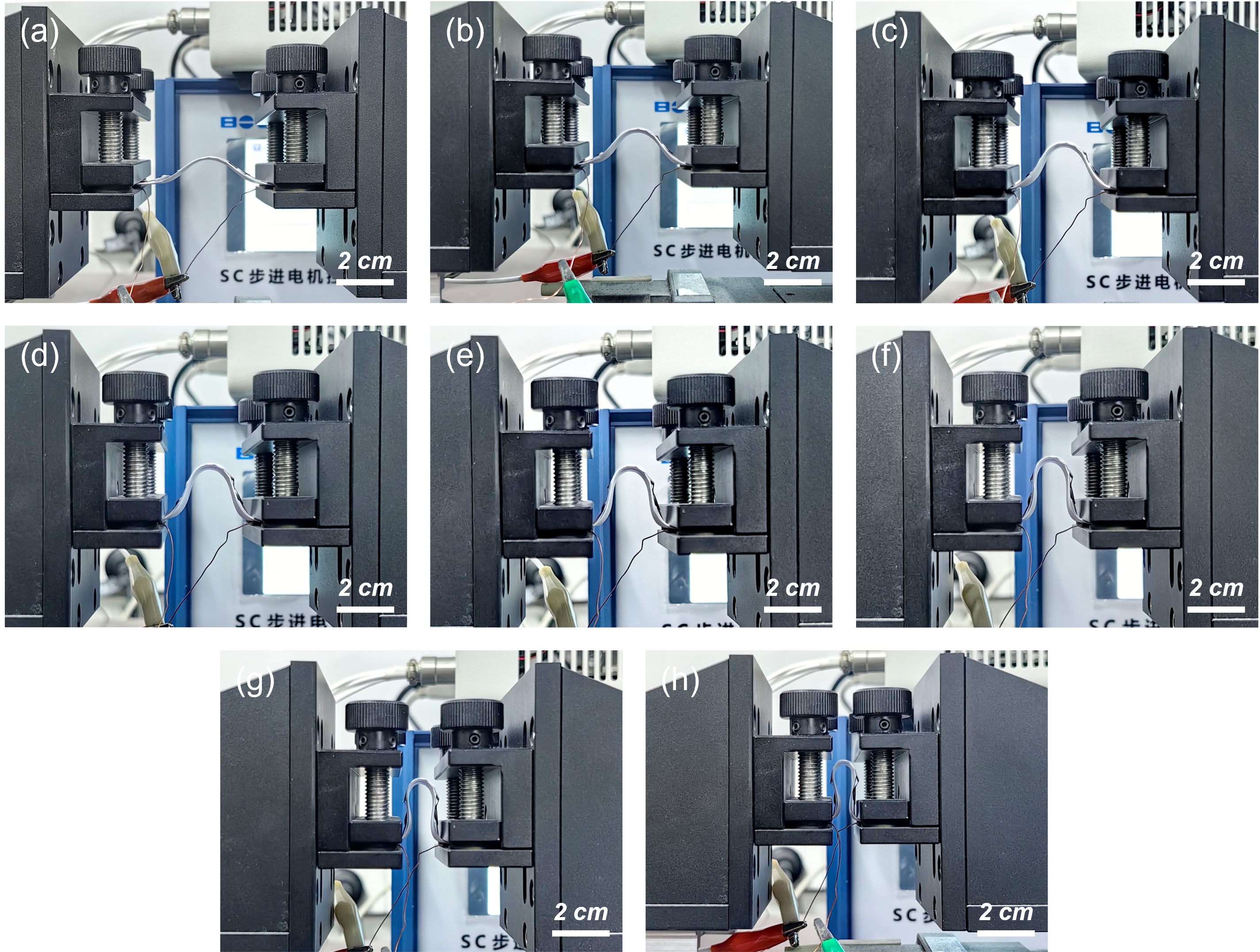


**Figure S15.** Optical images of the sensor based on BPNA/IL/BPNA composite film under bending deformations with varying displacements of (a) 5 mm, (b) 10 mm, (c) 15 mm, (d) 20 mm, (e) 25 mm, (f) 30 mm, (g) 35 mm and (h) 40 mm.


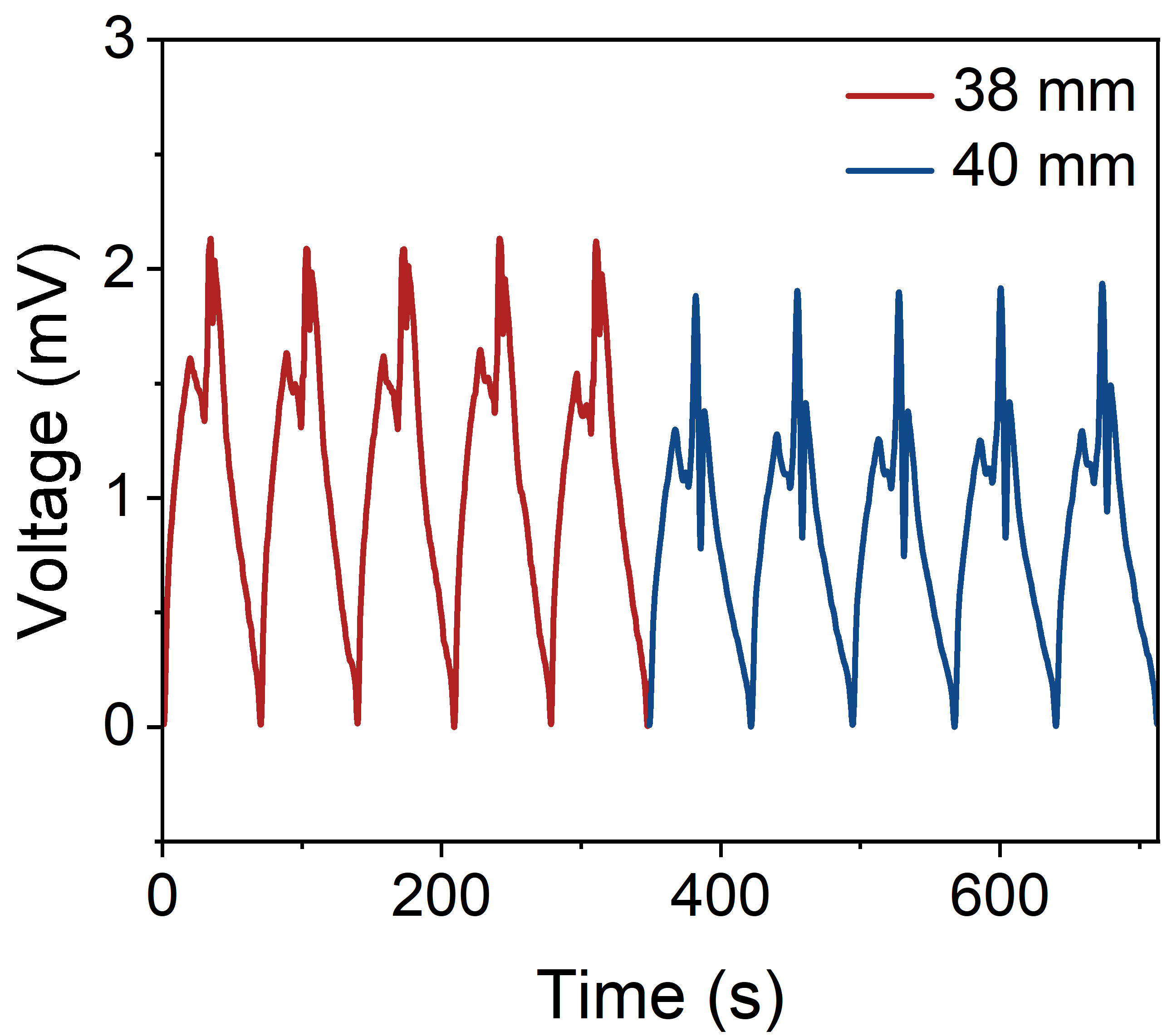


**Figure S16.** Sensing response of the sensor based on BPNA/IL/BPNA composite film under plastic bending deformations with displacements of 38 mm (0.60% strain) and 40 mm (0.62% strain).


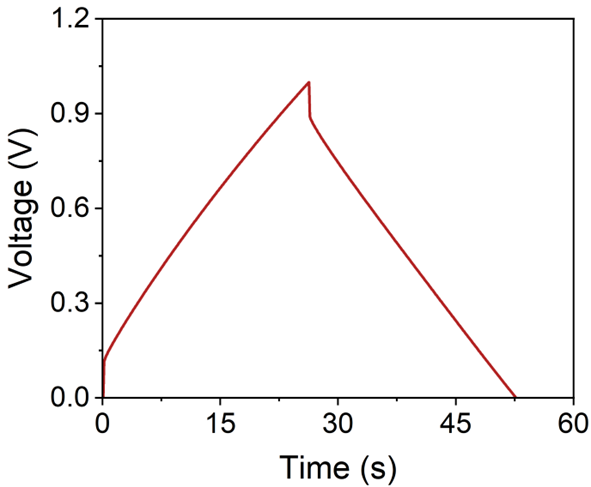


**Figure S17.** Charge-discharge curve of the sensor based on BPNA/IL/BPNA composite film at current density of 0.5 A g^-1^.


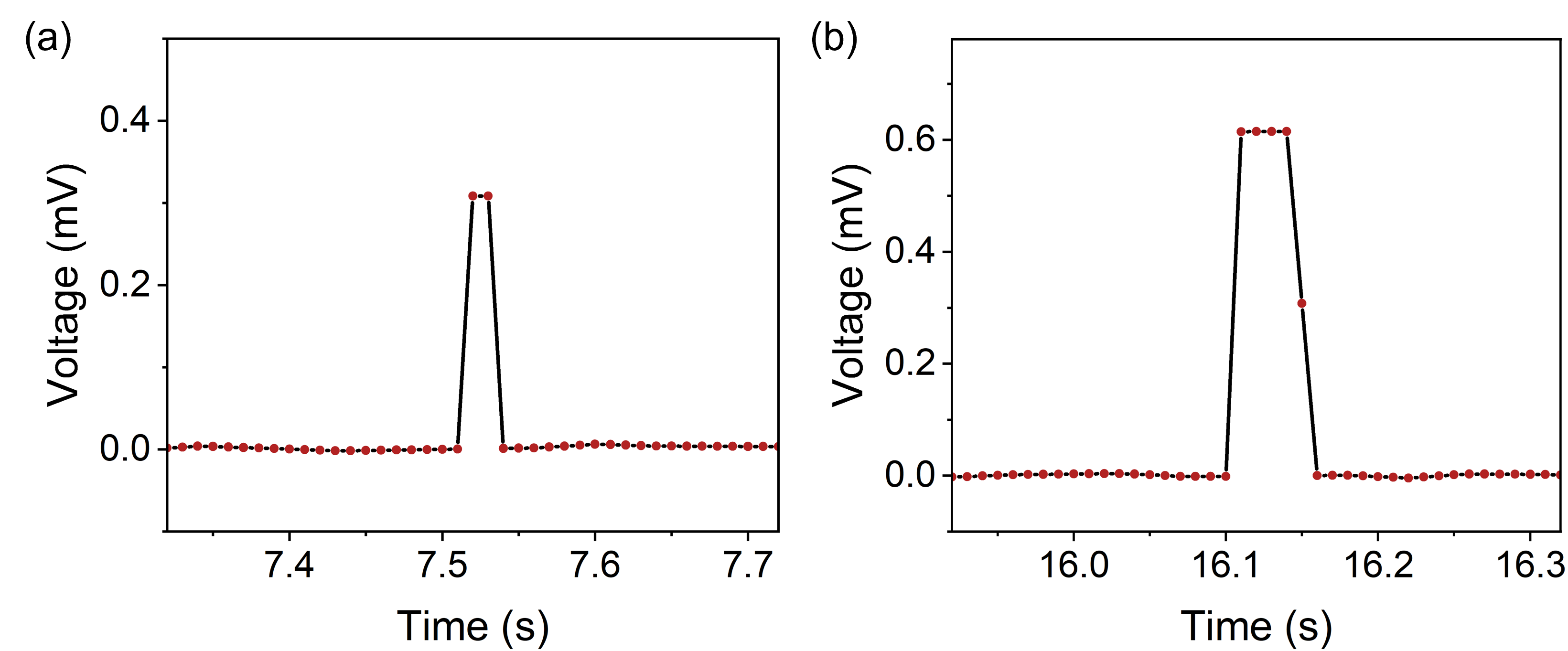


**Figure S18.** Response and recovery time tested by dropping (a) 10 g and (b) 40 g balls separately from a height of 10 mm onto the sensor surface.


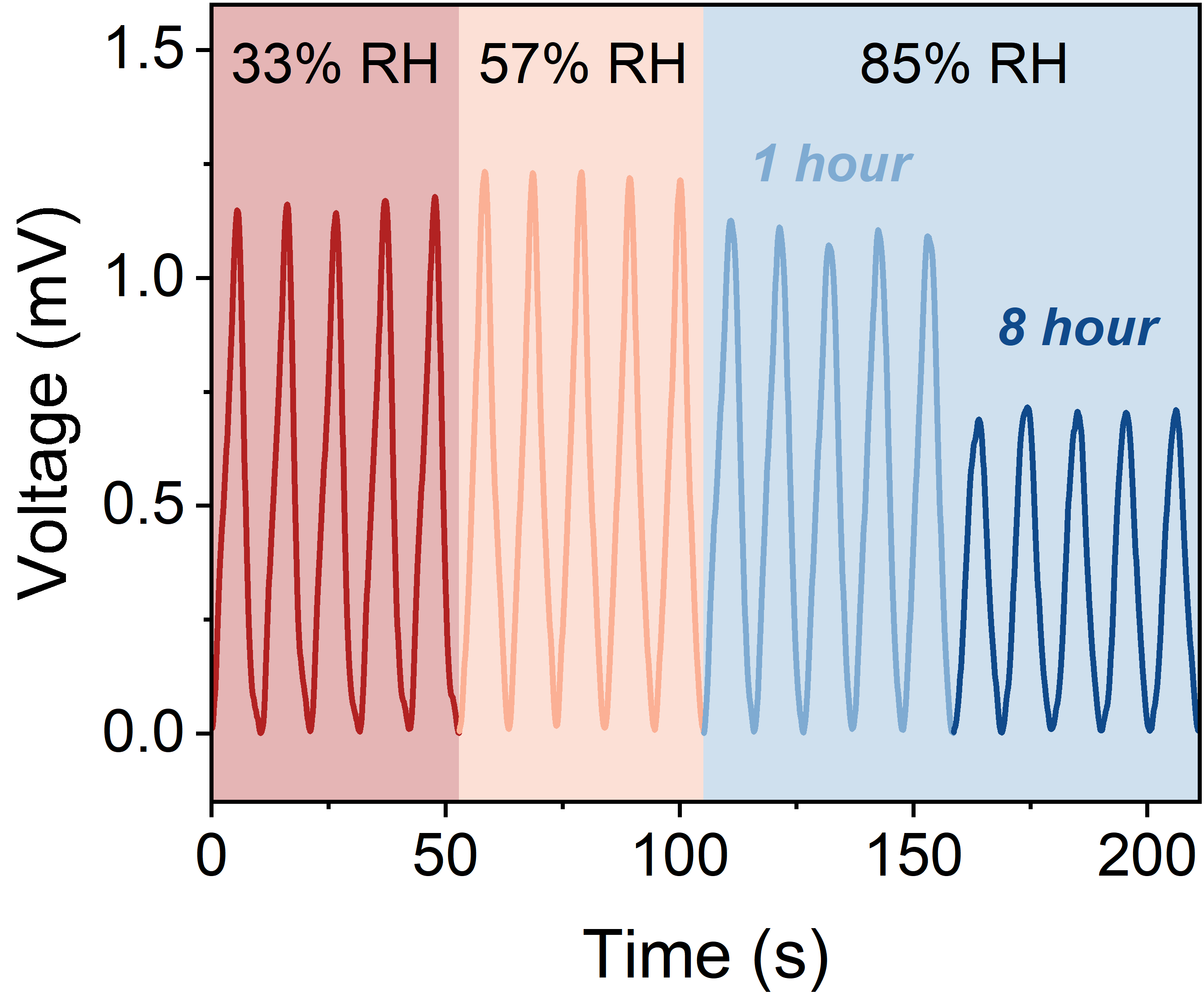


**Figure S19.** Comparison of voltage signals under varied humidity conditions: 33% RH, 57% RH and 85% RH (at 1 h and 8 h respectively).


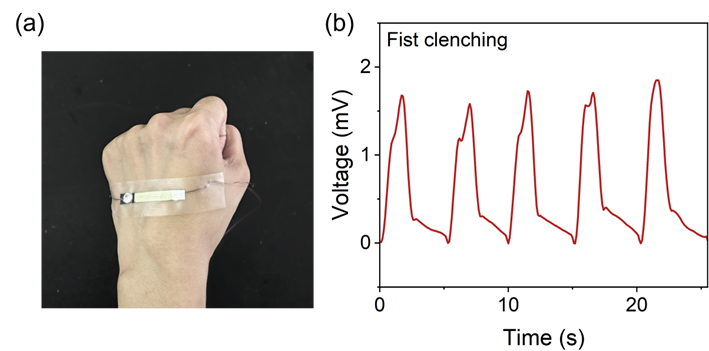


**Figure S20.** (a) Optical image of the sensor attached to the back of the hand. (b) Voltage output signals of fist clenching.


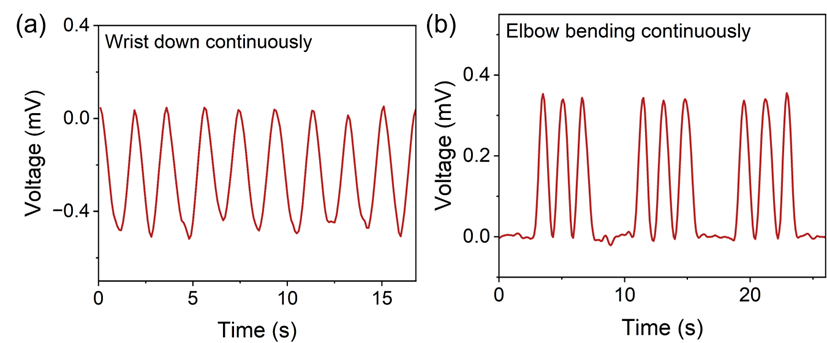


**Figure S21.** Voltage output signals of (a) consecutive downward wrist bends and (b) three consecutive elbow bends.


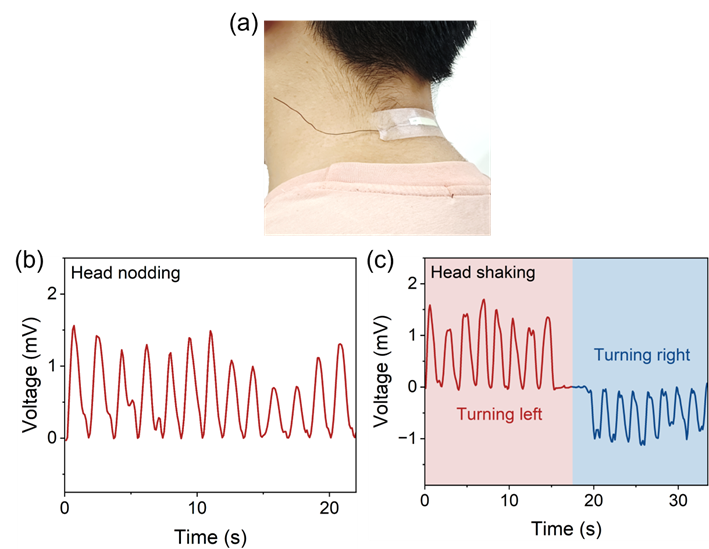


**Figure S22.** (a) Optical image of the sensor attached to the posterior cervical region. Voltage output signals of (b) head nodding and (c) head shaking in left and right directions.


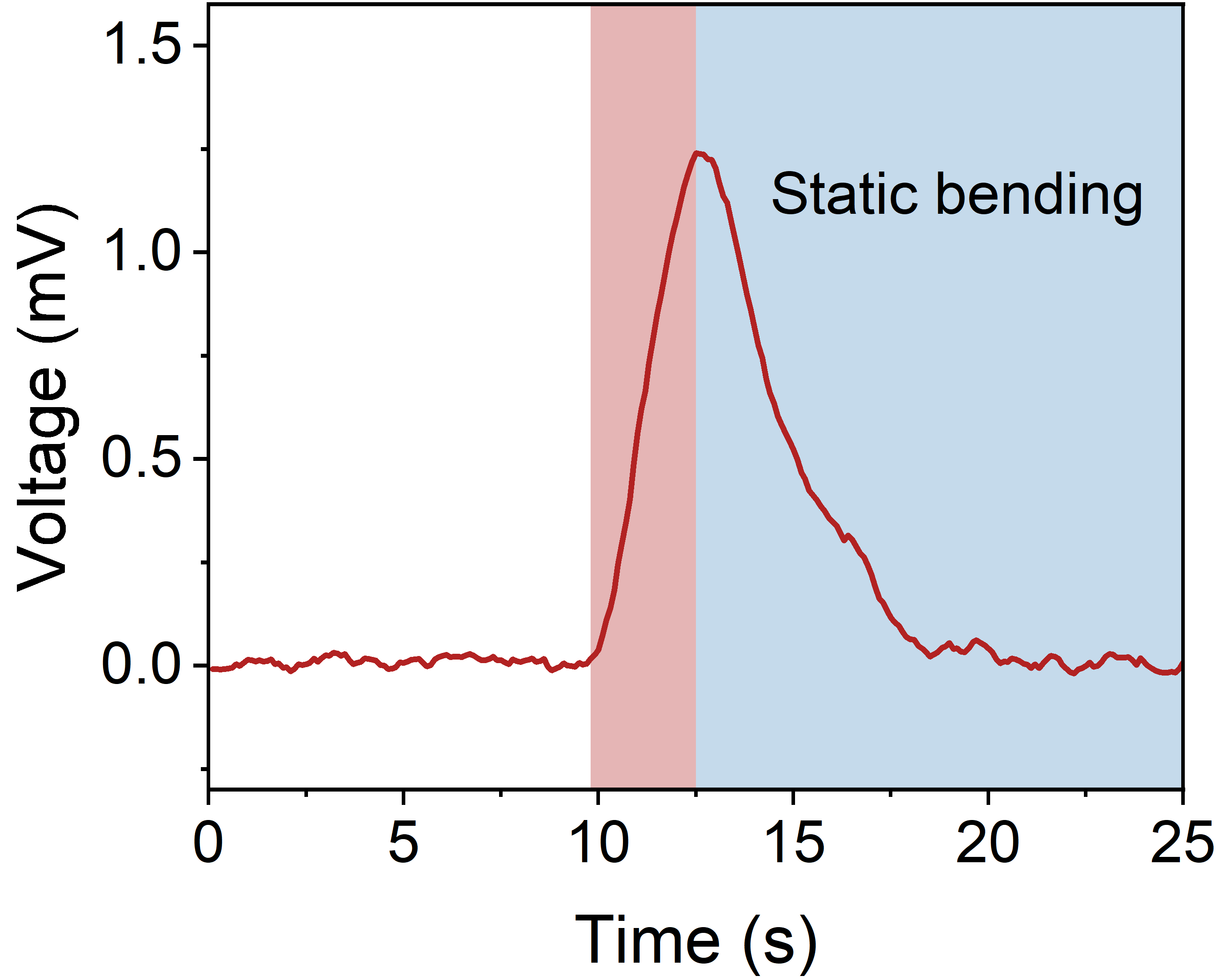


**Figure S23.** Sensing response of the sensor dynamic bending (red region) and under static bending with a constant strain (blue region).


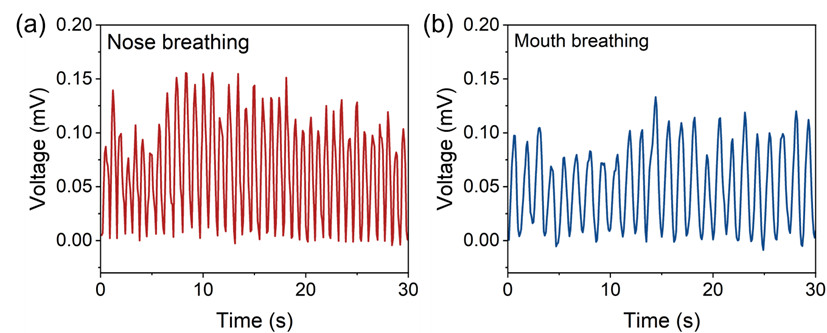


**Figure S24.** Voltage output signals with Tester 1 of (a) rapid nose breathing and (b) rapid mouth breathing.


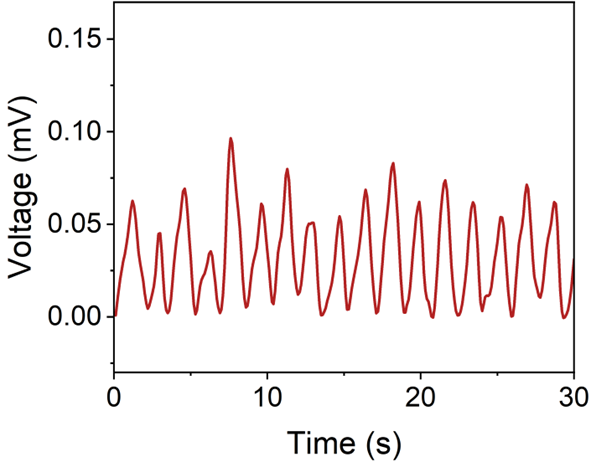


**Figure S25.** Respiratory monitoring with Tester 3.

**Table S1.** Values for all relevant parameters by fitting the EIS data with the equivalent circuit model.

|  | R-BPNA  (kΩ) | Q-BPNA  (F·s^α-1^) | α-BPNA | χ^2^ |
| --- | --- | --- | --- | --- |
| 10 wt% | 1.64 | 4.15E-8 | 0.698 | 0.022 |
| 15 wt% | 1.53 | 5.58E-8 | 0.666 | 0.019 |
| 30 wt% | 1.12 | 5.18E-8 | 0.803 | 0.064 |

|  | R-ionic  (Ω) | Q-ionic  (F·s^α-1^) | α-ionic |  |
| --- | --- | --- | --- | --- |
| 10 wt% | 895 | 6.01E-6 | 0.466 |  |
| 15 wt% | 606 | 4.71E-5 | 0.263 |  |
| 30 wt% | 784 | 1.01E-5 | 0.374 |  |

|  | R-EDL  (kΩ) | Q-EDL  (F·s^α-1^) | α-EDL |  |
| --- | --- | --- | --- | --- |
| 10 wt% | 331 | 3.12E-4 | 0.899 |  |
| 15 wt% | 6.18 | 1.35E-3 | 0.758 |  |
| 30 wt% | 86.8 | 4.86E-4 | 0.782 |  |

**Table S2.** Comparison of voltage signal of piezoionic strain sensors.

| Ref. | Strain (%) | Voltage (mV) |
| --- | --- | --- |
| 1 | 1.5 | 2.11 |
| 2 | 1 | 0.5 |
| 3 | 0.65 | 1.4 |
| 4 | 1.8 | 1.3 |
| 5 | 2 | 2.9 |
| This work | 0.51 | 3.28 |

**References**

[1] C. Lu, and X. Chen, “Piezoionic Strain Sensors Enabled by Force-Voltage Coupling from Ionogels,” *Chem. Phys. Lett.* 803 (2022): 139872.

[2] M. Li, J. Qiao, C. Zhu, et al., “Gel-Electrolyte-Coated Carbon Nanotube Yarns for Self-Powered and Knittable Piezoionic Sensors,” *ACS Appl. Electron. Mater.* 3 (2021): 944-954.

[3] J. Zhao, S. Han, Y. Yang, et al., “Passive and Space-Discriminative Ionic Sensors Based on Durable Nanocomposite Electrodes toward Sign Language Recognition,” *ACS Nano* 11 (2017): 8590-8599.

[4] Y. Liu, Y. Hu, J. Zhao, G. Wu, X. Tao, and W. Chen, “Self-Powered Piezoionic Strain Sensor toward the Monitoring of Human Activities,” *Small* 12 (2016): 5074-5080.

[5] A. Adjaoud, G. T. M. Nguyen, L. Chikh, et al., “Piezoionic Sensors Based on Formulated PEDOT:PSS and Aquivion for Ionic Polymer-Polymer Composites,” *Smart Mater. Struct.* 30 (2021): 105027.
